# Functional analyses of small secreted cysteine‐rich proteins identified candidate effectors in *Verticillium dahliae*

**DOI:** 10.1111/mpp.12921

**Published:** 2020-03-10

**Authors:** Dan Wang, Li Tian, Dan‐Dan Zhang, Jian Song, Shuang‐Shuang Song, Chun‐Mei Yin, Lei Zhou, Yan Liu, Bao‐Li Wang, Zhi‐Qiang Kong, Steven J. Klosterman, Jun‐Jiao Li, Jie Wang, Ting‐Gang Li, Sabiu Adamu, Krishna V. Subbarao, Jie‐Yin Chen, Xiao‐Feng Dai

**Affiliations:** ^1^ Laboratory of Cotton Disease Institute of Food Science and Technology Chinese Academy of Agricultural Sciences Beijing China; ^2^ Institute of Plant Protection Chinese Academy of Agricultural Sciences Beijing China; ^3^ College of Life Science Qufu Normal University Qufu China; ^4^ Key Laboratory of Agro‐products Quality and Safety Control in Storage and Transport Process Ministry of Agriculture Beijing China; ^5^ United States Department of Agriculture Agricultural Research Service Salinas CA USA; ^6^ Department of Plant Pathology University of California Davis, c/o United States Agricultural Research Station Salinas CA USA

**Keywords:** disulphide bonds, effector, immunity, pathogen‐associated molecular pattern (PAMPs), small cysteine‐rich proteins (SCPs), *Verticillium dahliae*, virulence

## Abstract

Secreted small cysteine‐rich proteins (SCPs) play a critical role in modulating host immunity in plant–pathogen interactions. Bioinformatic analyses showed that the fungal pathogen *Verticillium dahliae* encodes more than 100 VdSCPs, but their roles in host–pathogen interactions have not been fully characterized. Transient expression of 123 VdSCP‐encoding genes in *Nicotiana benthamiana* identified three candidate genes involved in host–pathogen interactions. The expression of these three proteins, VdSCP27, VdSCP113, and VdSCP126, in *N. benthamiana* resulted in cell death accompanied by a reactive oxygen species burst, callose deposition, and induction of defence genes. The three VdSCPs mainly localized to the periphery of the cell. BAK1 and SOBIR1 (associated with receptor‐like protein) were required for the immunity triggered by these three VdSCPs in *N. benthamiana*. Site‐directed mutagenesis showed that cysteine residues that form disulphide bonds are essential for the functioning of VdSCP126, but not VdSCP27 and VdSCP113. *VdSCP27*, *VdSCP113*, and *VdSCP126* individually are not essential for *V. dahliae* infection of *N. benthamiana* and *Gossypium hirsutum*, although there was a significant reduction of virulence on *N. benthamiana* and *G. hirsutum* when inoculated with the *VdSCP27*/*VdSCP126* double deletion strain. These results illustrate that the SCPs play a critical role in the *V. dahliae–*plant interaction via an intrinsic virulence function and suppress immunity following infection.

## Graphical abstract

Small cysteine‐rich proteins secreted by *Verticillium dahliae* play critical roles in interactions with hosts via an intrinsic virulence function and can suppress immunity following infection.

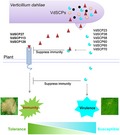

## 1 INTRODUCTION

Some fungi have evolved the ability to cause plant diseases and are a threat to many economically important crops. Although plants lack mobile defence cells or adaptive immune systems, they have evolved an innate immune system to recognize and respond to pathogens (Cook *et al.*, 2015). Innate immunity in plants involves the induction of cascading antimicrobial responses following recognition of a pathogen, including rapid changes in calcium ion levels, a reactive oxygen species (ROS) burst, callose deposition, cell wall reinforcement, and increases in defence‐related gene expression (Boller and Felix, 2009; Zipfel, 2009).

Plants can detect pathogens via different immunogenic signals: pathogen‐ or microbe‐associated molecular patterns (PAMP/MAMP) (Jones and Dangl, 2006; Zipfel, 2008). Recognition of PAMPs by plant cell surface‐localized pattern recognition receptors (PRRs) triggers defence responses known as PAMP‐triggered immunity (PTI). To surmount plant innate immunity, pathogens may deliver effector molecules either to the host apoplast or cytoplasm, resulting in effector‐triggered susceptibility (ETS). Pathogen effector proteins may also be recognized by plant R proteins, which activate a second layer of immunity known as effector‐triggered immunity (ETI) (Jones and Dangl, 2006; Zipfel, 2008; Dodds and Rathjen, 2010; Giraldo and Valent, 2013). From the point of view of a pathogen, secreted effectors play a critical role in successful infection through multiple pathways and especially by suppressing plant defence responses (Toruño *et al*., 2016).

Since the molecular cloning of the first fungal effector from *Cladosporium fulvum* (van Kan *et al.*, 1991), numerous effectors have been characterized and investigated in many plant pathogens (Houterman *et al*., 2008; Stergiopoulos and de Wit, 2009). Features that discriminate fungal effectors from secreted non‐effectors are predominantly sequence length, molecular weight, net charge, as well as cysteine, serine, and tryptophan content (Sperschneider *et al.*, 2016). The effectors generally present low sequence similarity to known proteins, domains, and motifs (Rep, 2005). Therefore, certain features are predictive of potential candidate effectors in fungi, including the presence of signal peptides, protein size, the lack of transmembrane domains or glycosyl‐phosphatidyl‐inositol (GPI)‐anchor sites, cysteine‐richness, and localization in the host plant (Rep, 2005; Stergiopoulos and de Wit, 2009; Kim *et al.*, 2016). In particular, small cysteine‐rich proteins (SCPs) are typical of the known apoplastic effectors (Rep, 2005; Stergiopoulos and de Wit, 2009; Lu and Edwards, 2016; Qi *et al.*, 2016; Cheng *et al.*, 2017) in which cysteine residues form disulphide bonds that appear to enhance effector stability in the apoplastic space, which is rich in proteases (Kamoun, 2006; Stergiopoulos and de Wit, 2009; Saunders *et al.*, 2012). For instance, *Rhynchosporium secalis Nip1* encodes a small protein with 10 cysteine residues and five intramolecular disulphide bridges (Rohe *et al.*, 1995). Small proteins that are rich in cysteine residues and predicted to be secreted have been proposed as likely candidates for effector proteins that play a critical role during host–pathogen interactions.

*Verticillium dahliae* is a notorious fungal pathogen that attacks a wide range of hosts, targets the xylem tissue, and causes verticillium wilt disease on many economically important crops (Klosterman *et al.*, 2009; Inderbitzin and Subbarao, 2014). Previous studies have shown that secreted proteins probably play a critical role in the pathogenesis of *V. dahliae* (Fradin and Thomma, 2006; Chu *et al.*, 2015; Chen *et al.*, 2016a; 2016b; Zhang *et al.*, 2018), and the function or the activity of several secreted proteins have been elucidated (de Sain and Rep, 2015; Klimes *et al.*, 2015; Wang *et al.*, 2018; Zhang *et al.*, 2018). Similar to the mechanisms discovered in other phytopathogens, *V. dahliae* secretes proteins to manipulate host immunity during infection (de Jonge *et al.*, 2012; Zhou *et al.*, 2012; Santhanam *et al.*, 2013; Liu *et al.*, 2014; Gui *et al.*, 2017, 2018; Kombrink *et al.*, 2017; Zhang *et al.*, 2017; Qin *et al.*, 2018). Many of these effectors are relatively small and enriched in cysteine residues, including Ave1, VdCBM1, and VdSCP7 (VDAG_07157) (de Jonge *et al.*, 2012; Gui *et al.*, 2017; Zhang *et al.*, 2017). For instance, the avirulence gene *Ave1* encodes an SCP preprotein of 134 amino acids and contains four cysteine residues in the mature protein (de Jonge *et al.*, 2012). Complete genome sequencing has greatly facilitated the rapid identification of genes encoding SCPs. Data mining of the *V. dahliae* secretome showed that the VdLs.17 genome encodes more than 100 hypothetical proteins that were designated as small (≤400 amino acids), cysteine‐rich (≥4 cysteine residues) proteins, and some of these potentially function as effectors (Klosterman *et al.*, 2011). Our recent study of the *V. dahliae* Vd991 (from *Gossypium hirsutum*) genome identified 127 SCPs (Chen *et al.*, 2018) but the functions of SCPs in *V. dahliae* have remained largely unknown.

In the current study, all 127 SCPs encoded in the genome of *V. dahliae* Vd991 were investigated for their activity in plant–pathogen interactions using a transient expression system in *Nicotiana benthamiana*. *N. benthamiana* was the system of choice for this work because it is fast growing, highly susceptible to *V. dahliae* (Wright and Biss, 1968), and a model host species to screen for gene functions through leaf agroinfiltration (Bally *et al.*, 2018).

The main objectives of this study were to (a) characterize the features of SCPs in *V. dahliae* Vd991; (b) determine specific SCPs that trigger an immune response in *N. benthamiana* using a transient expression assay; (c) study the potential role of disulphide bridges in SCP‐triggered immunity; (d) test whether other SCPs could suppress immunity; and (e) investigate the virulence function of three SCPs in cotton and *N. benthamiana* that could induce the immune responses.

## 2 RESULTS

### 2.1 Identification of genes encoding VdSCPs in the *V. dahliae* genome

**Figure 1:**
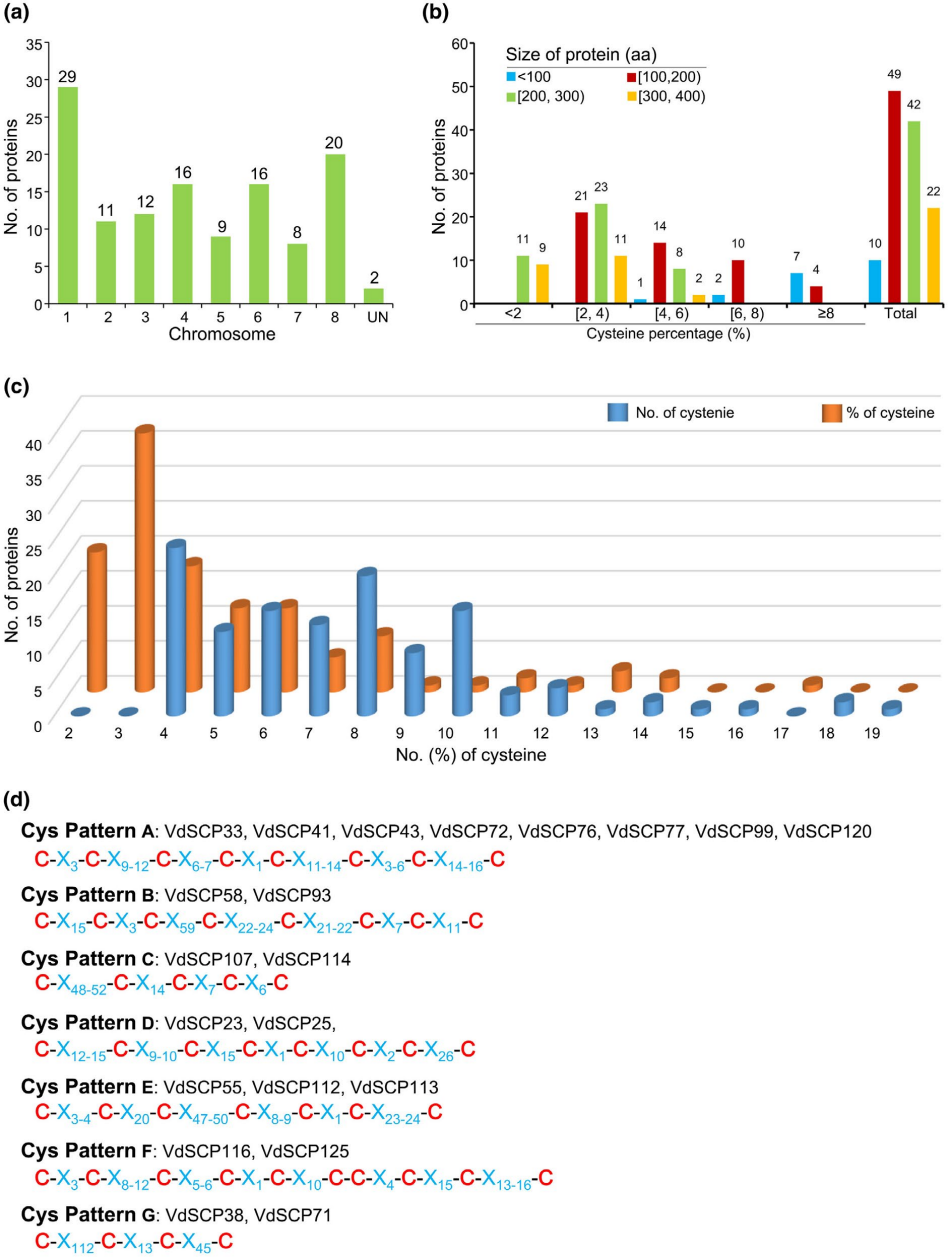
An overview of the small secreted cysteine‐rich proteins encoded in the genome of *Verticillium dahliae* (VdSCPs). (a) Analysis of the distribution of VdSCPs encoded in the eight chromosomes of *V. dahliae* strain Vd991. UN, two VdSCPs encoded in the assembled sequences that cannot mapped to the chromosomes of the *V. dahliae* strain JR2 reference genome (de Jonge *et al*., 2015). (b) Distribution of protein size and percentage of cysteine. The half open interval [...) represents the range of cysteine percentage, e.g. [2, 4) means the cysteine percentage is greater than or equal to 2% and less than 4%. (c) Calculation of protein numbers according to the number and percentage of cysteine residues, respectively. (d) Analysis of the conserved distribution pattern of the cysteine residues of the VdSCPs. X represents the random amino acid residue, and the subscript numbers represent the total number of amino acid residues between the two cysteines

In total 739 genes were predicted to encode secreted proteins in the genome of *V. dahliae* (strain Vd991). Among these, 127 hypothetical proteins encoding SCPs (<400 amino acids, ≥4 cysteine residues here described as VdSCPs) were predicted as effectors (Chen *et al.*, 2018). These VdSCPs contain a signal peptide but lack transmembrane domains (Table S1), typical characteristics of proteins transported to the extracellular space, where they may be involved in host–pathogen interactions. Four VdSCPs containing four cysteine residues (VdSCR3, VdSCR46, VdSCR57, and VdSCR59) were excluded from the set of predicted SCPs due to the presence of one cysteine residue within the signal peptide, which would result in mature proteins with only three cysteine residues (Tables S2 and S3). Physical location analysis showed that the VdSCPs are distributed across all eight chromosomes in Vd991 (Figure 1a and Table S3), and their delineation on the chromosomes from Vd991 was determined by chromosomal synteny with the reference genome of strain JR2 (Chen *et al.*, 2018). Chromosome 1 harbours the largest number (29 in total) of VdSCPs (Figure 1a and Table S3). Most VdSCPs are between 100 and 300 amino acids and contained 2%–4% cysteine residues (Figure 1b and Table S3). To further understand the characteristics of VdSCPs, the distribution pattern of cysteine residues for each protein was investigated by the interval length between the neighbouring cysteine residues, revealing that the cysteine residues in some VdSCPs have a similar distribution pattern (Figure 1c and Table S4). For instance, nine VdSCPs have similar distribution patterns of cysteine residues C‐X_3_‐C‐X_9‐12_‐C‐X_6‐7_‐C‐X_1_‐C‐X_11‐14_‐C‐X_4‐6_‐C‐X_14‐16_‐C (Figure 1c and Table S4), and are composed of the common domain (IPR008427, CFEM domain) in several fungal extracellular membrane proteins (Table S5). However, the distribution of cysteine residues in most VdSCPs (101 in total) did not follow this pattern. In addition, comparative analysis of VdSCPs to the SCPs encoded in the *V. dahliae* genomes from strains JR2 and VdLs.17 showed that 10 VdSCPs were specific to Vd991 (Table S3). Together, these results indicate that the *V. dahliae* genomes of different strains encode a variety of SCPs, which potentially play different roles during host infection.

### 2.2 VdSCP27, VdSCP113, and VdSCP126 display cell death‐inducing activities in *N. benthamiana*

**Figure 2:**
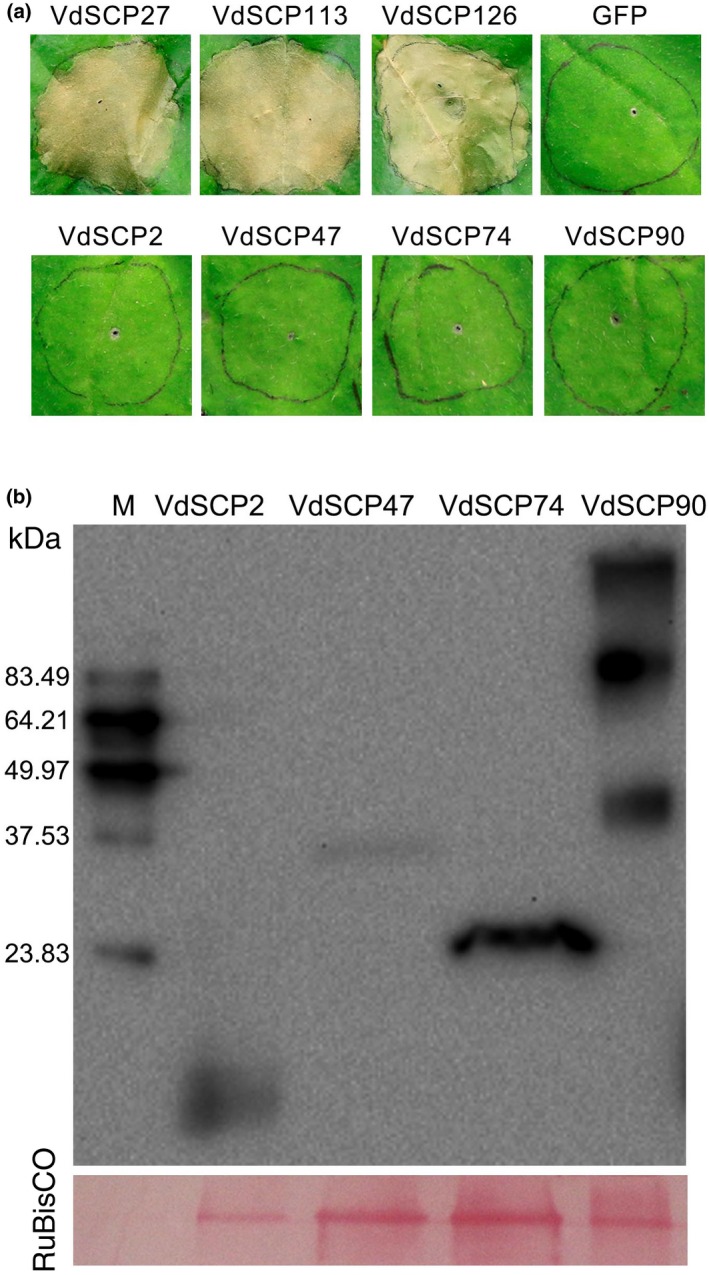
Identification of cell death‐inducing activity of *Verticillium dahliae* secreted small cysteine‐rich proteins (VdSCPs) in *Nicotiana benthamiana*. (a) Induction of cell death was detected for VdSCPs in *N. benthamiana* leaves from 4‐week‐old plants at 6 days after infiltration with *Agrobacterium tumefaciens* expressing the indicated genes. The construct expressing green flourescent protein (GFP) was used as a negative control. (b) Immunoblotting analysis of four non‐cell death‐inducing activity of VdSCPs (VdSCP1, VdSCP47, VdSCP74, and VdSCP90) fused to the FLAG‐tag in *N. benthamiana* leaves 60 hr after infiltration. Ponceau S‐stained RuBisCO protein is a total protein loading control

Cell death triggered by transient expression of VdSCPs associated with plant immune responses was investigated in *N. benthamiana* leaves. Among the 123 VdSCPs tested, only those encoded by *VdSCP27* (*VEDA_02400*), *VdSCP113* (*VEDA_09020*), and *VdSCP126* (*VEDA_09813*) triggered cell death in *N. benthamiana* leaves 6 days after agroinfiltration (Figures 2a and S1). Although immunoblotting analysis confirmed the effective translation of four additional arbitrarily selected VdSCPs (VdSCP2, VdSCP47, VdSCP74, and VdSCP90), these failed to trigger cell death in *N. benthamiana* (Figure 2a,b). These results indicate that VdSCP27, VdSCP113, and VdSCP126 in particular possess cell death‐inducing activities in *N. benthamiana*.

*VdSCP27*, *VdSCP113*, and *VdSCP126* encode proteins of 214, 191, and 212 amino acids in length, and contain four, seven, and four cysteine residues, respectively (Table S3). BlastP analysis revealed proteins with no known functions homologous to these three VdSCPs that encode hypothetical proteins, and only VdSCP27 contains a conserved domain (IPR025649, protein of unknown function DUF4360) as predicted by InterProScan (Table S5).

### 2.3 VdSCP27, VdSCP113, and VdSCP126 mainly localize on the periphery of *N. benthamiana* cells

**Figure 3:**
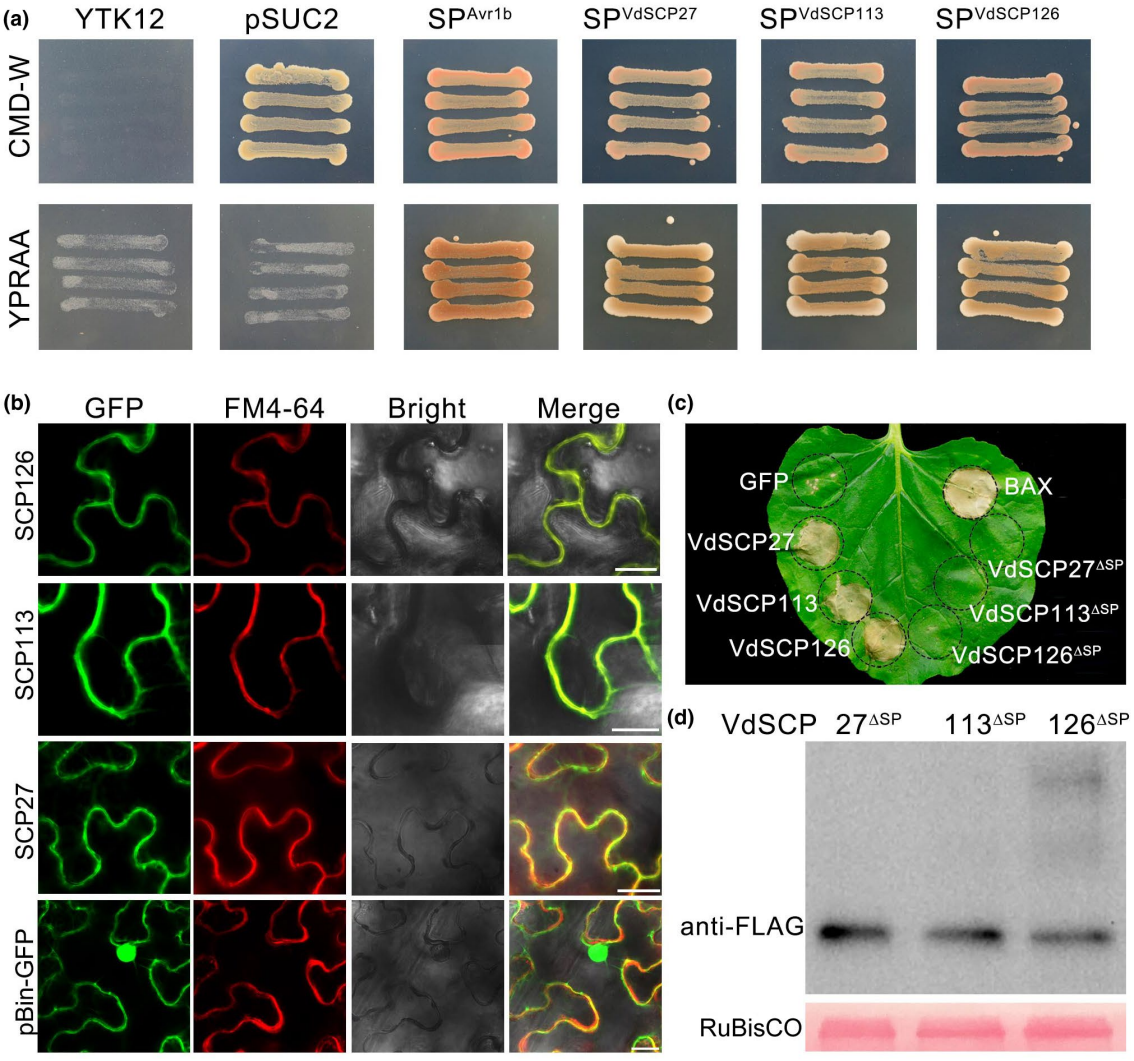
Subcellular localization of the *Verticillium dahliae* proteins VdSCP27, VdSCP113, and VdSCP126. (a) Functional validation of the putative N‐terminal signal peptide of VdSCP27, VdSCP113, and VdSCP126 using the yeast invertase secretion assay (Liu *et al*., 2013). The yeast strain YTK12 could not grow on CMD–W medium (i.e., without tryptophan). The strain containing the pSUC2 vector grew based on the function of the *Trp* operon. Fusion of the functional signal peptide of each of the three VdSCPs in‐frame with mature yeast invertase enabled secretion of invertase, resulting in growth on YPRAA medium. The known functional signal peptide Avr1b was used as a positive control. (b) Subcellular localization of VdSCP27, VdSCP113, and VdSCP126 was determined by transient expression of C‐terminal green fluorescent protein (GFP)‐tagged proteins in *Nicotiana benthamiana* leaves. The fluorochrome FM4‐64 was used for dyeing the cell membrane. Three genes were transiently expressed in 4‐week‐old *N. benthamiana* leaves and harvested at 2 days after agroinfiltration. The vector pBin‐GFP was used as negative control. Fluorescence was scanned using a Leica TCS SP8 confocal microscopy system using 200× magnification with an excitation wavelength at 488 nm and emission at 510 nm for GFP, an excitation wavelength at 543 nm and emission of 562 nm for FM4‐64. Bars = 35 μm. (c) Functional signal peptides are required for cell death‐inducing activities of VdSCP27, VdSCP113, and VdSCP126 in *N. benthamiana*. Deletion of the signal peptide encoding region in *VdSCP27*, *VdSCP113*, and *VdSCP126* (yielding the products VdSCP27^∆SP^, VdSCP113^∆SP^, and VdSCP126^∆SP^) resulted in no induction of cell death in 4‐week‐old plants at 6 days after infiltration. Positive and negative control constructs expressed Bcl‐2‐associated X protein (BAX) and GFP, respectively. (d) Immunoblotting analysis of transiently expressed VdSCP27^∆SP^, VdSCP113^∆SP^, and VdSCP126^∆SP^ fused to the FLAG‐tag in *N. benthamiana* leaves at 60 hr after infiltration. The expected sizes of VdSCP27^∆SP^, VdSCP113^∆SP^, and VdSCP126^∆SP^ were 21.2, 17.9, and 20.4 kDa, respectively. Ponceau S‐stained RuBisCO protein is a total protein loading control

To test the secretory function of VdSCP27, VdSCP113, and VdSCP126, the yeast signal trap assay system based on the requirement of yeast cells for invertase secretion to grow on sucrose or raffinose media was used for the functional analysis of the putative N‐terminal signal peptide (Liu *et al.*, 2013; Table S1). Like the known functional signal peptide Avr1b, the signal peptide of all three VdSCPs had the ability to mediate the secretion of invertase after each signal peptide region was fused with the invertase (Gu *et al*., 2011) (Figure 3a), indicating that VdSCP27, VdSCP113, and VdSCP126 are indeed secreted proteins*.*

VdSCP27, VdSCP113, and VdSCP126 induced cell death in *N. benthamiana* (Figure 2a), revealing that these three VdSCPs are recognized by the host. To detect the subcellular location of VdSCP27, VdSCP113, and VdSCP126, the coding regions of each of these VdSCPs were fused with green fluorescent protein (GFP) and transiently expressed in *N. benthamiana* leaves by agroinfiltration. Using the cell membrane dye FM4‐64, the results showed that the three native VdSCPs with the signal peptide aggregated at the periphery of *N. benthamiana* cells, including along plasma membrane, though some GFP signal was present in the cytoplasm, such as the cytosolic bridges (Figure 3b). Interestingly, transient expression of the three VdSCPs lacking the signal peptide (VdSCP27^ΔSP^, VdSCP113^ΔSP^, and VdSCP126^ΔSP^) induced fluorescence signals mainly concentrated along the periphery of *N. benthamiana* cells in a particle‐like form, and many fluorescent particles appeared inside the plant cell (Figure S2). Furthermore, they also did not induce cell death 6 days post‐agroinfiltration in *N. benthamiana* (Figure 3c), although immunoblotting analysis indicated that all of the VdSCPs lacking the signal peptide in *N. benthamiana* were correctly translated (Figure 3d). In addition, these results suggest that the extracellular secretion mediated by the N‐terminal signal peptide and distribution along the periphery of *N. benthamiana* cell is required for VdSCPs to induce cell death. Together, these results suggest that VdSCP27, VdSCP113, and VdSCP126 are associated primarily with the cell periphery and act as effectors to induce a cell death response in the interaction with *N. benthamiana*.

### 2.4 VdSCP27, VdSCP113, and VdSCP126 trigger immunity in *N. benthamiana* by inducing a diversity of defence responses

**Figure 4:**
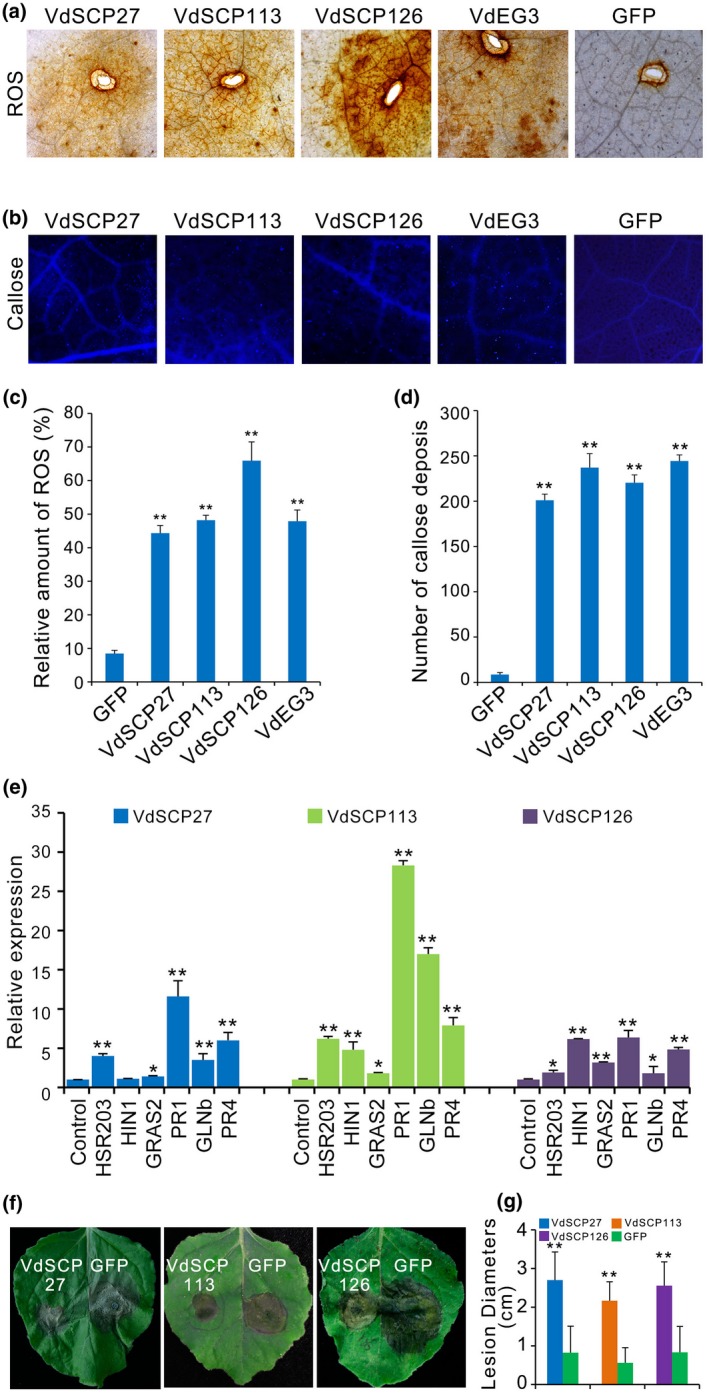
Identification of the immunity triggered by VdSCP27, VdSCP113, and VdSCP126 in *Nicotiana benthamiana*. (a) Reactive oxygen species (ROS) accumulation after transient expression of *VdSCP27*, *VdSCP113*, and *VdSCP126* in *N. benthamiana* leaves. ROS accumulation was assessed in *N. benthamiana* leaves from 4‐week‐old plants expressing *VdSCP27*, *VdSCP113,* and *VdSCP126* by staining with 3,3′‐diaminobenzidine. Expression of the known pathogen‐associated molecular pattern (PAMP) VdEG3 and green fluorescent protein (GFP) were included as positive and negative controls, respectively. (b) Detection of callose deposition by aniline blue straining. *N. benthamiana* leaves from 4‐week‐old plants were analysed 2 days after agroinfiltration with expression constructs encoding VdSCP27, VdSCP113, and VdSCP126. VdEG3 and GFP were used for positive and negative control constructs, respectively. (c) Detection of ROS induction levels associated with expression of VdSCP27, VdSCP113, and VdSCP126 after infiltration in *N. benthamiana*. Values are means ± *SD* from three independent experiments. Asterisks indicate a significant difference (*p* < .01) relative to the control of agroinfiltrated GFP expression construct in an unpaired Student's *t* test. (d) Average number of callose deposits/mm^2^ on expressing *VdSCP27*, *VdSCP113*, and *VdSCP126*. Values represent the means ± *SE* of three independent samples. Asterisks indicate a significant difference (*p* < .01) relative to the control of agroinfiltration GFP according to an unpaired Student's *t* test. (e) The induction defence response genes triggered by expression of *VdSCP27*, *VdSCP113*, and *VdSCP126*. The transcripts were detected by quantitative reverse transcription PCR in 4‐week‐old *N. benthamiana* leaves 24 hr after agroinfiltration of constructs encoding VdSCP27, VdSCP113, and VdSCP126, and GPF as control. *HSR203* (AF212184.1), *HIN1* (Y07563.1), *GRAS2* (KJ767660.1), *PR1* (AF480488.1), *GLNb* (M59442.1), *PR4* (AF154635.1). (f) Defence response induced by recombinant VdSCP27, VdSCP113, and VdSCP126 proteins. *N. benthamiana* leaves from a 5‐week‐old plant were pretreated with 100 mM of the indicated recombinant protein and inoculated 12 hr later with 5 µl of 2 × 10^6^ *B. cinerea* conidia/ml. Lesions were observed at 2 days post‐inoculation (dpi). (g) Lesion development of *B. cinerea* on *N. benthamiana* leaves was evaluated beginning 2 dpi by determining the average lesion diameter on six leaves from six plants each. Error bars represent standard errors of the mean. Values represent the averages of three independent measurements with three replicates each. Error bars represent standard errors of the mean. Asterisks (∗) and double asterisks (∗∗) represent statistical significance at *p* < .05 and *p* < .01, respectively, based on unpaired Student's *t* tests

To examine whether VdSCP27, VdSCP113, and VdSCP126 act as effectors to induce defence responses during host–pathogen interactions, several types of defence responses were assayed in *N. benthamiana* leaves following agroinfiltration, including the ROS accumulation, callose deposition, activation of defence response genes, and restriction of disease development. ROS accumulation was substantially enhanced 3 days after agroinfiltration of the constructs encoding VdSCP27, VdSCP113 or VdSCP126, and the known PAMP VdEG3 (Gui *et al.*, 2017) compared to the control agroinfiltrated with the GFP constructs (Figure 4a,c). *N. benthamiana* leaves displayed strong callose deposition after agroinfiltration with constructs encoding VdSCP27, VdSCP113 or VdSCP126 and the known PAMP VdEG3, compared to the control GFP (Figure 4b,d). Furthermore, several genes associated with immunity were significantly induced after agroinfiltration with the constructs encoding VdSCP27, VdSCP113 or VdSCP126, including hypersensitive response genes (*HSR203* and *HIN1*), the PTI‐associated marker gene *GRAS2*, and defence response genes associated with the salicylic acid signalling pathway (*PR1* and *GLNb*) and the jasmonic acid/ethylene signalling pathway (*PR4*) (Figure 4e). In contrast, GFP could not induce the expression of resistance genes compared with the positive control VdEG3 (Figure 4c). In addition, agroinfiltration of the *VdSCP27*, *VdSCP113* or *VdSCP126* constructs into *N. benthamiana* leaves resulted in a significant reduction of lesion area 48 hr after inoculation with *Botrytis cinerea*, compared to agroinfiltration of GFP alone (Figure 4f,g). Together, these results suggested that VdSCP27, VdSCP113, and VdSCP126 can each act as elicitors that induce cell death by triggering defence responses during host–pathogen interaction in *N. benthamiana*.

### 2.5 BAK1 and SOBIR1 are required for VdSCP27‐, VdSCP113‐, and VdSCP126‐triggered immunity in *N. benthamiana*

**Figure 5:**
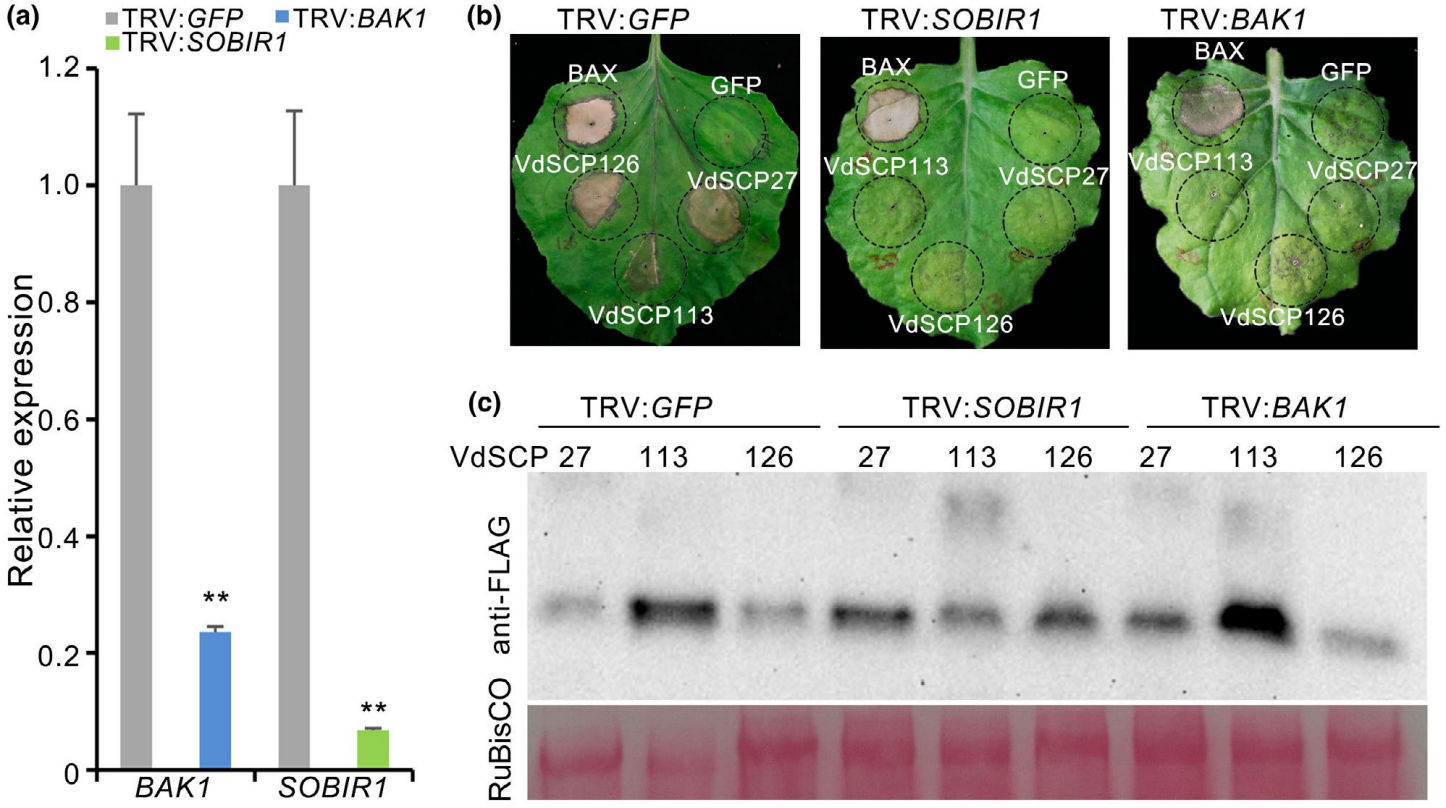
Relationship between VdSCP27, VdSCP113, VdSCP126, and the *Nicotiana benthamiana* proteins BAK1 and SOBIR1. (a) The silencing efficiency of *BAK1* and *SOBIR1* in *N. benthamiana* was determined by quantitative reverse transcription PCR, and *EF‐1a* of *N. benthamiana* was used as an endogenous control. Means and standard errors from three biological replicates are shown. Double asterisks represent statistical significance at *p* < .01 based on unpaired Student's *t* tests. (b) Validation of BAK1 or SOBIR1 involved in cell death induced by VdSCP27, VdSCP113, and VdSCP126 in *N. benthamiana. VdSCP27*, *VdSCP113*, and *VdSCP126* were transiently expressed in *BAK1‐* and *SOBIR1*‐silenced plants that were subjected to virus‐induced gene silencing by inoculation with tobacco rattle virus (TRV) constructs for 3 weeks. Green fluorescent protein (GFP)‐silenced plants served as negative controls. (c) Immunoblotting analysis of VdSCP27, VdSCP113, and VdSCP126 protein fused to a FLAG‐tag transiently expressed in *BAK1*‐ and *SOBIR1*‐silenced *N. benthamiana* leaves 60 hr after agroinfiltration. Ponceau S‐stained RuBisCO protein is a total protein loading control

BRI1‐associated receptor kinase1 (BAK1) and Suppressor of BIR1‐1 (SOBIR1) act as co‐receptor proteins that play important roles in disease resistance by interacting with PAMPs to trigger immunity, and are classified as a leucine‐rich repeat receptor‐like protein kinase (LRR‐RLK) and a leucine‐rich repeat receptor‐like protein (LRR‐RLP), respectively (Monaghan and Zipfel, 2012; Liebrand *et al.*, 2013; Zhang *et al.*, 2013). To test whether VdSCP27, VdSCP113, and VdSCP126 function autonomously on BAK1 and/or SOBIR1 in eliciting PAMP‐induced cell death, we generated virus‐induced gene silencing (VIGS) constructs based on recombinant tobacco rattle virus (TRV) to target *NbBAK1* and *NbSOBIR1* expression in *N. benthamiana*. The transcript levels of *NbBAK1* and *NbSOBIR1* were significantly reduced to 24% and 7% of the control level (Figure 5a), respectively. As expected, VdSCP27, VdSCP113, and VdSCP126 failed to trigger cell death in the *NbBAK1*‐silenced plants, while the positive control Bcl‐2‐associated X protein (BAX) retained cell death‐inducing activity (Figure 5b). Similarly, all of the three *VdSCP* constructs failed to induce cell death in *NbSOBIR1*‐silenced plants (Figure 5b). Immunoblotting analysis confirmed that VdSCP27, VdSCP113, and VdSCP126 were successfully expressed in the areas of infiltration in the gene‐silenced plants (Figure 5c). These results suggest that BAK1 and SOBIR1, which generally form the LRR‐RLP/SOBIR1/BAK1 complex, are required for VdSCP27, VdSCP113, and VdSCP126 to trigger immunity in *N. benthamiana*.

### 2.6 Cysteine residues are essential for the function of VdSCP126, but not VdSCP27 and VdSCP113

**Figure 6:**
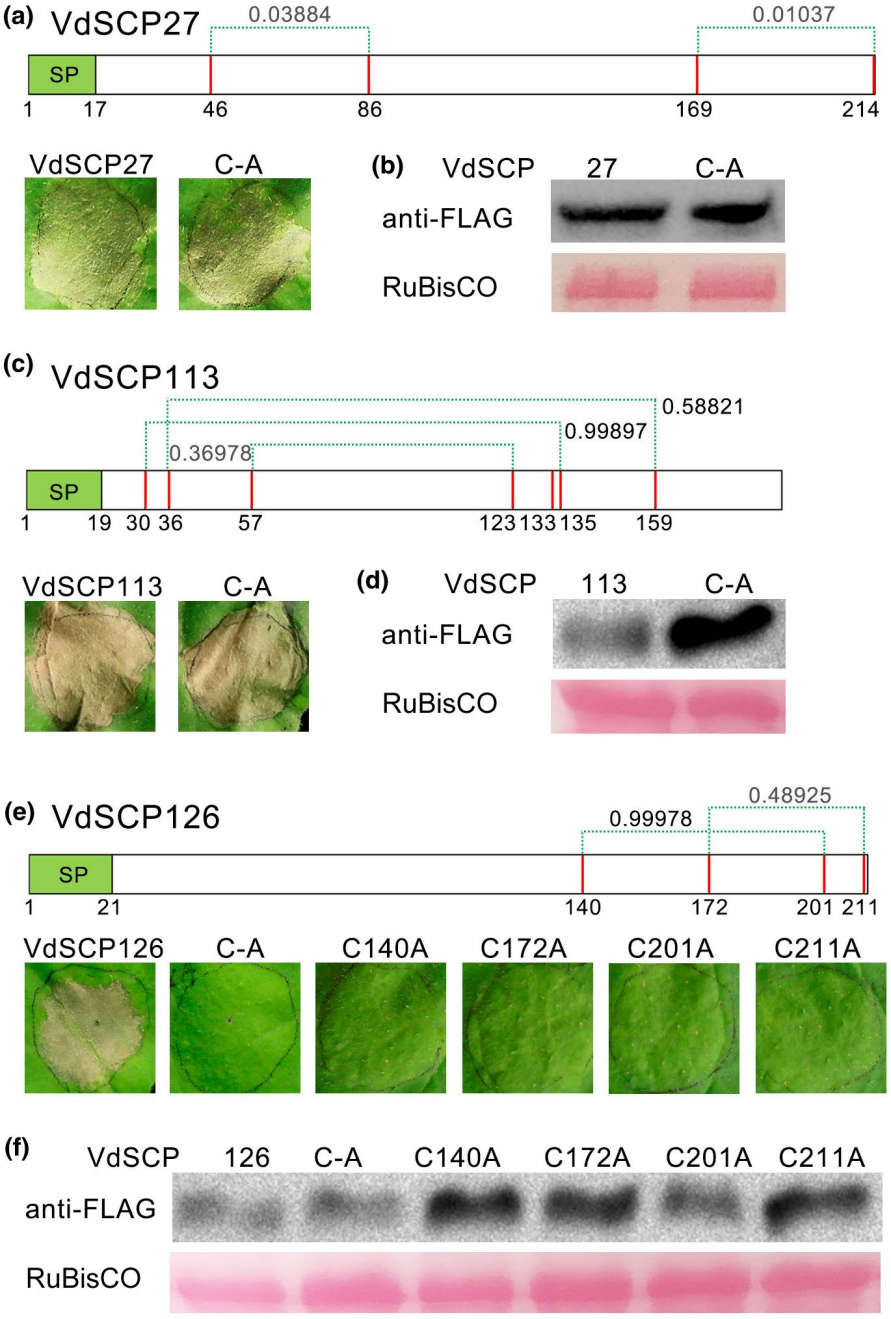
Prediction and functional analysis of disulphide bonds in the *Verticillium dahliae* proteins VdSCP27, VdSCP113, and VdSCP126. Disulphide bond predictions for VdSCP27 (a), VdSCP113 (c), and VdSCP126 (e) were conducted using the web‐based program DiANNA (http://clavius.bc.edu/~clotelab/DiANNA/). Values represent the score of cysteine residues likelihood of involvement in disulphide bond formation. SP, signal peptide. (a), (c), (e) The function of cysteine residues and the importance of disulphide bond formation were detected by cell death induction assays in 4‐week‐old *Nicotiana benthamiana* leaves after agroinfiltration of wild‐type genes and cysteine residue mutant alleles. The leaf phenotypes were photographed 6 days after agroinfiltration. “C‐A” indicates all cysteine residues were replaced by alanine; “C‐No.‐A” indicates the single cysteine residues were replaced by alanine in the respective position in VdSCPs. The efficiency of transient expression of the wild‐type genes and cysteine residue mutant alleles encoding the *V. dahliae* proteins VdSCP27 (b), VdSCP113 (d), and VdSCP126 (f) were validated by immunoblotting analysis 60 hr after agroinfiltration.in *N. benthamiana*. Ponceau S‐stained RuBisCO protein is shown as a total protein loading control

VdSCP27, VdSCP113, and VdSCP126 are enriched in cysteine residues that may be involved in disulphide bond formation. Bioinformatic analysis predicted two, three, and two cysteine disulphide bonds in VdSCP27, VdSCP113, and VdSCP126 (Figure 6), respectively, although the scores were relatively low for confirmation of some of these disulphide bonds. To test whether the cysteine residues in VdSCP27, VdSCP113, and VdSCP126 were essential to trigger immunity, mutant alleles of disulphide bonds in these VdSCPs were generated in which all cysteine residues were replaced with alanine (C‐A). Transient expression of these mutant alleles demonstrated that substitution of all cysteine residues in VdSCP27 and VdSCP113 retained cell death‐inducing activity in *N. benthamiana* but led to a complete loss of cell death‐inducing activity in the VdSCP126 mutant allele (Figure 6a,c,e). In addition, when single cysteine residues (C140A, C172A, C201A or C211A) were substituted in VdSCP126, the corresponding mutant allele did not induce cell death for up to 5 days after agroinfiltration in *N. benthamiana* leaves (Figure 6e). Immunoblotting analysis confirmed that all the mutant alleles of these three VdSCPs were successfully expressed 3 days after infiltration in *N. benthamiana* leaves (Figure 6b,d,f). These results suggested that cysteine residues are critical for inducing cell death in VdSCP126, but not in VdSCP27 and VdSCP113. We therefore speculate that the disulphide bonds probably are essential for the biological function of VdSCP126 during host–pathogen interactions, but the position of disulphide bonds that confers this function requires further scrutiny.

### 2.7 *V. dahliae* employs VdSCP effectors to suppress immunity triggered by VdSCP27, VdSCP113, and VdSCP126

**Figure 7:**
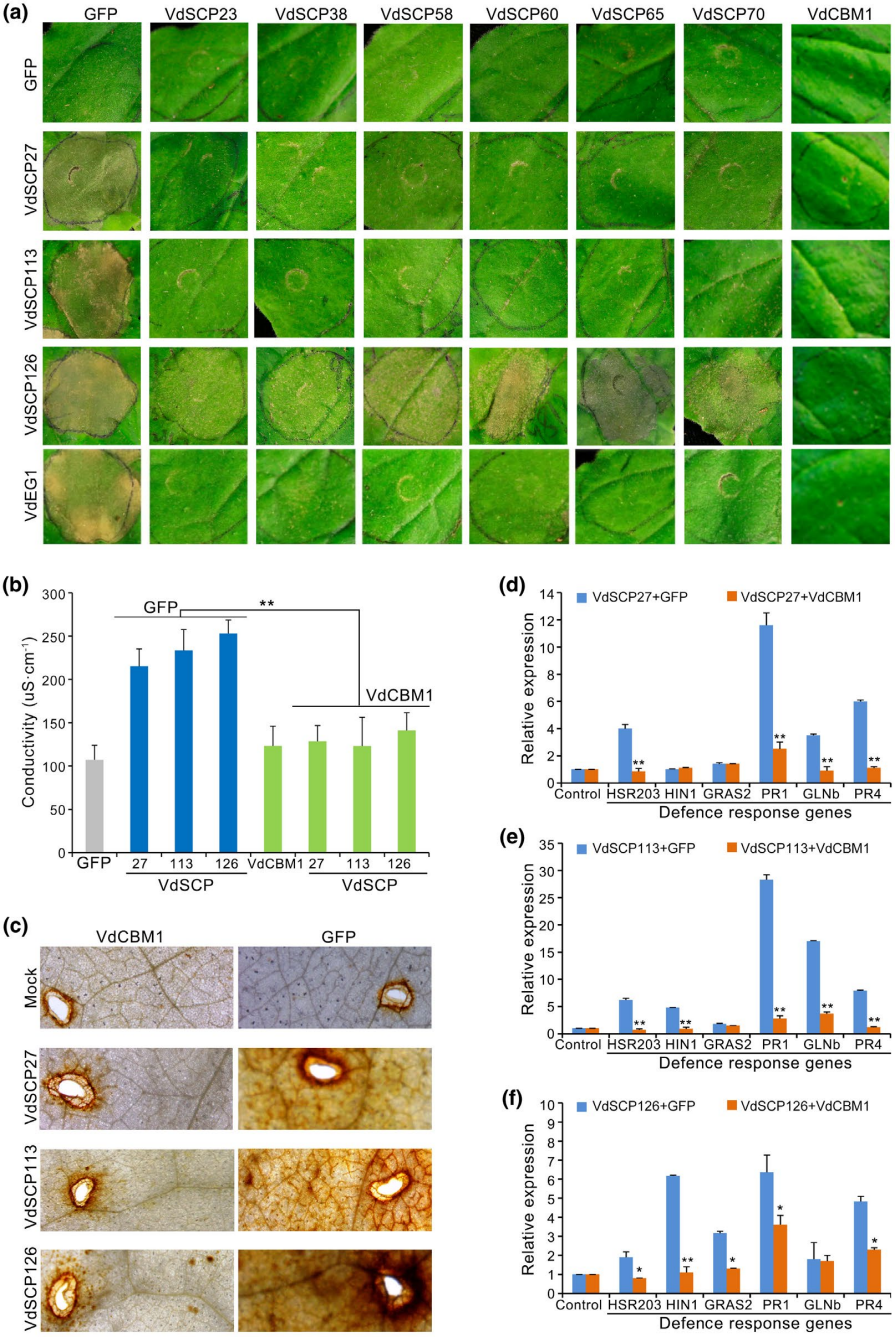
Suppression of VdSCP27, VdSCP113, and VdSCP126 cell death‐inducing activity by the *Verticillium dahliae* protein VdCBM1. (a) Assay for suppression of cell death‐inducing activity of six VdSCP effectors and VdCBM1 by co‐agroinfiltration with expression constructs encoding VdSCP27, VdSCP113, and VdSCP126 in 4‐week‐old *Nicotiana benthamiana* leaves. Co‐agroinfiltration of constructs encoding six VdSCP effectors. VdCBM1 and green fluorescent protein (GFP) were expressed for controls. Photographs were taken 6 days after agroinfiltration. (b) Electrolyte leakage assay in 4‐week‐old *N. benthamiana* leaves 48 hr after co‐infiltration of constructs expressing *VdSCP27*, *VdSCP113*, and *VdSCP126* simultaneously with *VdCBM1*. (c) 3,3′‐diaminobenzidine (DAB) staining of the inhibition of reactive oxygen species accumulation 6 days after co‐agroinfiltration of the *VdCBM1* construct with those of *VdSCP27*, *VdSCP113*, and *VdSCP126*. Co‐agroinfiltration of VdSCPs alone and GFP were used as controls. Agroinfiltration of empty vector (pGR107) served as a control (Mock). Quantitative reverse transcription PCR analyses of the inhibition of defence response gene expression 3 days after co‐agroinfiltration of expression constructs *VdCBM1* with *VdSCP27* (d), *VdSCP113* (e), and *VdSCP126* (f). Co‐agroinfiltration of VdSCPs alone and GFP were used as controls. Blue columns represent expression of defence‐related genes in response to each of the VdSCPs. Red columns represent the expression of defence‐related genes when VdSCPs were co‐infiltrated with VdCMB1. Values represent the averages of three independent measurements with three replicates each, and error bars represent standard errors of the mean. Asterisks (∗) and double asterisks (∗∗) represent statistical significance at *p* < .05 and *p* < .01, respectively, according to unpaired Student's *t* tests

To surmount immunity triggered by VdSCP27, VdSCP113, and VdSCP126, *V. dahliae* probably delivers other SCP effectors to promote plant susceptibility by suppressing plant immune responses. To test this hypothesis in *V. dahliae*, we selected a set of potential VdSCP effectors (VdSCP23, VdSCP38, VdSCP58, VdSCP60, VdSCP65, and VdSCP70) that suppressed cell death induced by the PAMP VdEG1 (unpublished data), and which trigger cell death and PTI in *N. benthamiana* (Gui *et al.*, 2017). All six VdSCP effectors that could suppress VdEG1‐triggered cell death also suppressed cell death triggered by VdSCP27 and VdSCP113 (Figure 7a), but could not completely suppress cell death triggered by VdSC126 (Figure 7a). VdCBM1 is a known effector that can suppress PTI during infection in *V. dahliae* (Gui *et al.*, 2017). We further tested whether VdCBM1 had the ability to suppress the immunity triggered by VdSCP27, VdSCP113, and VdSCP126. VdSCP27, VdSCP113, and VdSCP126 were each separately expressed with VdCBM1 in *N. benthamiana* by co‐agroinfiltration. Expression of VdCBM1 suppressed the immune response when compared to the normal cell death‐inducing activities associated with constructs encoding VdSCP27, VdSCP113, and VdSCP126 (Figure 7a). The VdCBM1‐encoding protein also contains four cysteine residues and was characterized previously as a SCP (Klosterman *et al.*, 2011; Gui *et al.*, 2017). These results suggested that cell death triggered by VdSCP27, VdSCP113, and VdSCP126 is differentially and effectively suppressed by VdSCP effectors in *N. benthamiana*. In addition, conductivity assays revealed that co‐expression of *VdSCP27*, *VdSCP113*, and *VdSCP126* separately with the GFP expression cassette induced electrolyte leakage 3 days after co‐agroinfiltration in *N. benthamiana* leaves, but the electrolyte leakage was significantly reduced in the areas of co‐agroinfiltration with *VdSCP27*, *VdSCP113*, and *VdSCP126* constructs in combination with the *VdCBM1* construct (Figure 7b). Correspondingly, the defence genes induced by the expression of VdSCP27, VdSCP113, or VdSCP126 were significantly suppressed when co‐expressed with VdCBM1 in *N. benthamiana* leaves (Figure 7d,e,f). In addition, ROS accumulation in *N. benthamiana* leaves was significantly impacted with co‐expression of VdCBM1 (Figure 7c).

### 2.8 *VdSCP27* and *VdSCP126* double deletion mutants display impaired virulence function during *N. benthamiana* and cotton infection

Investigation of the transcript levels of *VdSCP27*, *VdSCP113*, and *VdSCP126* revealed that each of these were significantly up‐regulated during infection on *N. benthamiana* and cotton plants (Figure S3a,b), suggesting that these three VdSCPs may play an important role in host–pathogen interactions. To explore the possible contributions of VdSCP27, VdSCP113, and VdSCP126 to pathogenicity and virulence, we generated gene deletion strains by replacing the coding sequences of the three *VdSCP*s with a hygromycin resistance cassette. Several independent deletion strains were verified by PCR (Figure S4), and two positive strains were selected for infection experiments with *N. benthamiana* and cotton plants using a root‐dip method. The pathogenicity assays showed that deletion of *VdSCP27*, *VdSCP113* or *VdSCP126* did not change the virulence toward *N. benthamiana* 21 days after inoculation, when verticillium wilt symptoms of necrosis and wilting are typically seen (Figure S6a). Quantification of fungal biomass by quantitative PCR in inoculated *N. benthamiana* revealed similar fungal biomass development with the deletion strain and the wild‐type strains (Figure S6c). Similarly, inoculation of *VdSCP27*, *VdSCP113* or *VdSCP126* deletion strains on susceptible cotton displayed the same verticillium wilt symptoms of necrosis, wilting, vascular discoloration, and fungal biomass development as the wild‐type strain Vd991 (Figure S6b,d).

**Figure 8:**
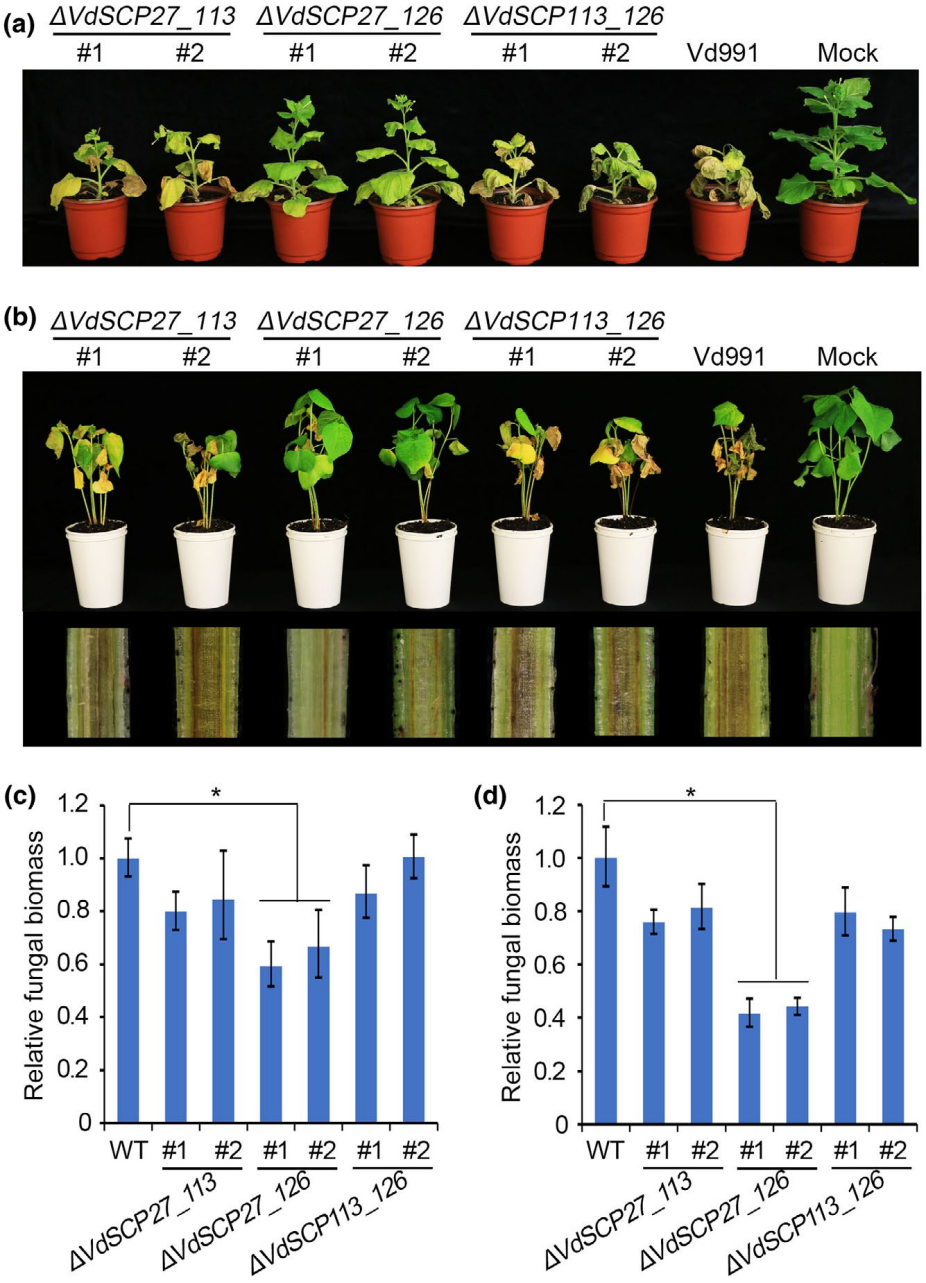
Analyses of virulence functions of double deletion mutant strains ∆*VdSCP27_113*, ∆*VdSCP27_126*, and ∆*VdSCP113_126* in *Nicotiana benthamiana* and *Gossypium hirsutum* interactions. (a) Phenotypes of *N. benthamiana* plants inoculated with the double gene deletion mutants of ∆*VdSCP27_113*, ∆*VdSCP27_126*, and ∆*VdSCP113_126*. Four‐week‐old seedlings of *N. benthamiana* plants were inoculated with respective double gene deletion strains with two independent transformations for each gene, in addition to inoculation with wild‐type *Verticillium dahliae* (Vd991) and sterile water (Mock). Verticillium wilt symptoms were photographed 3 weeks after inoculation. (b) Pathogenicity assays to investigate the role of double gene deletion mutant strains ∆*VdSCP27_113*, ∆*VdSCP27_126*, and ∆*VdSCP113_126* in virulence on *G. hirsutum* 'Junmian No. 1'. Relative fungal biomass of the double mutant strains ∆*VdSCP27_113*, ∆*VdSCP27_*126, and ∆*VdSCP113_126* on (c) *N. benthamiana* and (d) *G. hirsutum* was calculated in planta at 3 weeks post‐inoculation by quantitative PCR. Error bars represent standard errors of the mean. Asterisks (∗) represent statistical significance at *p* < .05 based on unpaired Student's *t* tests

To further detect whether *VdSCP27*, *VdSCP113* or *VdSCP126* were functional or exhibit quantitative redundancy during infection of host plants, double deletion mutants of two VdSCP pairs (∆*SCP27_113*, ∆*SCP27_126*, ∆*SCP113_126*) were constructed (Figure S5). The pathogenicity assays showed that deletion of *VdSCP27* and *VdSCP126* together impaired virulence toward both *N. benthamiana* and cotton plants (Figure 8a,b). Quantification of fungal biomass in inoculated *N. benthamiana* and cotton also revealed obvious fungal biomass reduction in deletion strain ∆*SCP27_126* compared with the wild‐type strains (Figure 8c,d). However, the other double deletion mutants (∆*SCP27*_*113* and ∆*SCP113*_*126*) did not impair the virulence towards the two hosts. These results indicate that *VdSCP27* and *VdSCP126* also act together as virulence factors in *N. benthamiana* and cotton interactions with *V. dahliae*.

## 3 DISCUSSION

In *V. dahliae*, more than 100 SCPs have been identified through comparative genomics approaches (Klosterman *et al.*, 2011; de Jonge *et al.*, 2012; Chen *et al.*, 2018), but only a few of these are known to truly participate in the pathogen infection process (de Jonge *et al*., 2012; Zhang *et al.*, 2017). In the current study, investigation of a large‐scale transient expression of nearly all *VdSCP*s that occur in strain Vd991 from cotton was undertaken. This involved the screening of 123 putative small cysteine‐rich secreted proteins (Chen *et al.*, 2018) in *N. benthamiana*. Only three SCPs, VdSCP27, VdSCP113, and VdSCP126, which act as PAMPs, were identified, and BAK1 and SOBIR1 (generally forming the LRR‐RLP/SOBIR1/BAK1 complex) are required for these VdSCPs to induce cell death and trigger immunity in *N. benthamiana*. To surmount these defence responses, *V. dahliae* employs other VdSCP effectors to suppress the immunity triggered by VdSCP27, VdSCP113, and VdSCP126. In addition, double deletion of *VdSCP27* and *VdSCP126* significantly impaired the virulence of *V. dahliae* in both *N. benthamiana* and cotton plants, indicating that they also act as virulence factors in *V. dahliae*. To our knowledge, this study is the first to systematically investigate all SCPs in a strain at the whole genome scale.

Comparative genomics analyses have suggested that *V. dahliae* secretes over 700 proteins, including more than 100 SCPs with unknown function (hypothetical protein) that are thought to function in the host apoplast to promote disease (Klosterman *et al.*, 2011; Chen *et al.*, 2018). In our previous study, we found that the genome of *V. dahliae* strain Vd991 encodes 127 SCPs of unknown function (Chen *et al.*, 2018), and confirmed that 123 encode SCPs based on their mature proteins (Table S3). With the large‐scale transient expression of these 123 VdSCPs, we first identified three new SCPs, VdSCP27, VdSCP113, and VdSCP126, that induced cell death in *N. benthamiana* (Figures 1 and S1). VdSCP27, VdSCP113, and VdSCP126 localized along the *N. benthamiana* cell periphery, possibly acting as PAMPs to induce cell death depending on their signal transduction by the BAK1/SOBIR1 complex (Figures 3 and 5). However, the subcellular location also showed that the three VdSCPs, especially VdSCP27 and VdSCP113, can also be located in cytoplasm, such as on the cytosolic bridges (Figure 3), suggesting that these three effectors also may have an intracellular role or are internalized similar to the effector SsSSVP1 (Lyu *et al.*, 2016). Only the *A. tumefaciens* pBin‐GFP was used as a negative control along with a cell membrane colocalizing dye. Additional controls that accumulate in the same cell compartments with target proteins (VdSCPs) could enhance our understanding of the accuracy of the subcellular locations of the three VdSCPs in future studies.

One obvious and common feature of SCPs, as well as some effectors that maintain structural stability in oxidizing environments, is the presence of multiple cysteine residues and their disulphide bonds (Sevier and Kaiser, 2002). The intramolecular disulphide bond is presumed to be important for protein folding, stability, and protection of such proteins in the harsh acidic and protease‐rich apoplast when they are delivered during plant infection (Kamoun, 2006). Disulphide bonds that are important for protein function have been identified in several effectors, including Avr2, Avr4, and Avr9 of *Cladosporium fulvum* (van den Hooven *et al.*, 2001; Luderer *et al*., 2002; van den Burg *et al.*, 2003; Van't Klooster *et al*., 2011), SsSSVP1 from *Sclerotinia sclerotiorum* (Lyu *et al.*, 2016), SnTox1 produced by *Stagonospora nodorum* (Liu *et al.*, 2012), and Pep1 from *Ustilago maydis* (Doehlemann *et al.*, 2009). In some cases, the post‐translational modification is also believed to be important for biological activity of SCPs, as in the modification of cysteine residues by S‐nitrosylation (Ling *et al.*, 2017). In the current study, mutational analysis showed that all cysteine residues are required for VdSCP126 to trigger cell death and immunity in *N. benthamiana* (Figure 6), suggesting that disulphide bonds formed by all cysteine residues probably are also important for the host infection mediated by VdSCP126. Unexpectedly, substitution of all cysteine residues in VdSCP27 and VdSCP113, however, did not affect the ability of each to induce cell death in *N. benthamiana* (Figure 6). The immunoblotting of SCP47 and SCP90 revealed that the actual sizes did not match the theoretical sizes, and these changes probably were caused by post‐translational modifications (Figure 2b). A few studies have characterized the lack of involvement of SCP cysteine residues in host–pathogen interactions. The role of cysteines residues in VdSCP27 and VdSCP113 will require further clarification.

During the coevolution of host–microbe interactions, pathogens acquired the ability to deliver effector proteins to interfere with PTI, enabling pathogens to infect their respective host plants and cause disease (Chisholm *et al.*, 2006; Jones and Dangl, 2006). For instance, *Puccinia striiformis* secretes a candidate effector, PSTha5a23, which mediates plant defence suppression and promotes wheat rust (Cheng *et al.*, 2017). Similarly, the immunity triggered by VdSCP27, VdSCP113, and VdSCP126 in a BAK/SOBIR1‐dependent manner can also be suppressed by several VdSCP effectors (Figure 6a). In our previous study, we found that *V. dahliae* can deliver the effector VdCBM1, a protein that also contains four cysteine residues and is a secreted SCP that suppresses PTI (Gui *et al.*, 2017). VdCBM1 can also suppress the immunity triggered by damage‐associated molecular patterns (DAMPs), including the products from VdCUT11 (Gui *et al.*, 2018). In this study, we rediscovered that the immunity triggered by VdSCPs in a BAK1/SOBIR1‐dependent manner is also suppressed by VdCBM1 (Figures 5 and 7). These results indicate that the *V. dahliae* VdSCPs could be part of a network that participates in host–pathogen interaction to surmount the immunity triggered by VdSCPs and promote colonization during infection.

Some SCPs are required for full virulence in many pathogens that have been analysed (Bolton *et al*., 2008; Doehlemann *et al.*, 2009; Chen *et al.*, 2016b; Qin *et al.*, 2018). In our study, we found that deletion of *VdSCP27*, *VdSCP113*, or *VdSCP126* independently did not affect the virulence of *V. dahliae* in tobacco and cotton plants (Figure S4). These results conflicted with our initial expectation that the immunity triggered by these VdSCPs is eliminated in the corresponding gene deletion strains, resulting in enhanced virulence during host infection, as deletion of immunity factor *VdSCP7* (*VDAG_07157*) significantly enhanced virulence on cotton (Zhang *et al.*, 2017). Interestingly, analyses of the double *VdSCP* deletion mutant showed that *VdSCP27* and *VdSCP126* together act as virulence factors in *V. dahliae* (Figure 8). In *Phytophthora sojae*, glycoside hydrolase 12 protein is a major virulence factor during soybean infection and is recognized as a PAMP (Ma *et al*., 2015), and so are VdEG1 and VdEG3 from *V. dahliae* (Gui *et al.*, 2017), suggesting that VdSCP27 and VdSCP126 function in virulence, although they also may be recognized by the host plants to induce immunity. Some fungal effectors are often considered dispensable for virulence due to functional or quantitative redundancy (Sharpee and Dean, 2016), as many PAMPs have been found in *V. dahliae* (de Jonge *et al.*, 2012; Zhou *et al.*, 2012; Santhanam *et al.*, 2013; Gui *et al.*, 2017; 2018) and deletion of VdSCP113 singly perhaps is unable to alter the pathogen virulence. Another hypothesis is that the effectors initially function in virulence and subsequently are recognized by the host to induce immunity as a result of a long coevolutionary arms race, thereby also forcing a dual role during host–pathogen interactions. This may explain the finding that the double deletion mutant could reduce virulence, whereas the single gene deletion mutant did not reduce virulence, but can induce immunity.

In conclusion, through a high‐throughput screen of all VdSCPs, our study demonstrated that transiently expressed VdSCP27, VdSCP113, and VdSCP126 in *N. benthamiana* participate in host–pathogen interactions. These three VdSCPs act as PAMPs to induce PTI responses differentially in a BAK1‐dependent or BAK/SOBIR1‐dependent manner, and cysteine residues are essential for the function of VdSCP126. *V. dahliae* probably employs the effector VdCBM1 and other six VdSCP effectors to surmount the immunity triggered by these three VdSCPs. Genes encoding VdSCP27, VdSCP113, and VdSCP126 are individually not essential for *V. dahliae* infection of host plants, but simultaneous deletion of both VdSCP27 and VdSCP126 contributes to reduced virulence on host plants. These results illustrate that the SCPs play a critical role through the intrinsic virulence function and suppress plant immunity functions. However, some of these have apparently been selected during the course of evolution of *V. dahliae–*host interactions and are thus recognized by the host to induce immunity.

## 4 EXPERIMENTAL PROCEDURES

### 4.1 Growth of microbial and plant material

The *V. dahliae* wild‐type strain Vd991 (highly virulent isolate from *G. hirsutum*) was cultured on potato dextrose agar (PDA) or in liquid Czapek medium for 7 days at 25 °C. The *V. dahliae* genetic transformants constructed in this study were cultured on PDA with 50 μg/ml hygromycin B. A *B. cinerea* wild‐type strain was grown on PDA at 20 °C. *Agrobacterium tumefaciens* GV3101 was cultured in Luria Bertani medium (tryptone 10 g, NaCl 10 g, yeast extract 5 g, water 1 L) at 28 °C for fungal transformations and transient expression experiments in plants. Cotton (*G. hirsutum* 'Junmian No. 1') was grown at 28 °C for 2 weeks for pathogenicity assays. *N. benthamiana* plants were grown at 25 °C for 4 weeks for pathogenicity assays and transient expression experiments. Both *G. hirsutum* and *N. benthamiana* were grown with a 14‐hr light/10‐hr dark photoperiod in a greenhouse at the temperatures noted above.

### 4.2 Bioinformatics analysis of VdSCPs

Sequences of the VdSCPs in this study were derived from the SCP predictions using the strain Vd991 reference genome (Chen *et al.*, 2018). DNA sequences corresponding to each of the chromosomes of strain Vd991 were assembled based on alignment with the *V. dahliae* strain JR2 genome (de Jonge *et al.*, 2013; Chen *et al.*, 2018). Subcellular localization predictions of VdSCPs were performed using the Fungi model of WoLF PSORT (Horton *et al.*, 2007) to identify the extracellular secretion characteristics, the signal peptides and signal peptide cleavage sites of VdSCPs were predicted using SignalP v. 4.1 (D‐score cut‐off set to 0.500) (Petersen *et al.*, 2011), and the transmembrane domains were predicted by Phobius (Käll *et al.*, 2007) and TMHMM 2.0 (Krogh *et al.*, 2001). The conserved motifs of VdSCPs were analysed with the InterPro database (Apweiler *et al.*, 2001). The orthologue (allelic) relationships among *V. dahliae* Vd991, JR2 and VdLs.17 were determined with BLASTP (Altschul *et al.*, 1997). Identification of disulphide bonds in VdSCPs was performed using DiANNA (http://clavius.bc.edu/~clotelab/DiANNA/).

### 4.3 Plasmid construction and preparation

For transient expression in *N. benthamiana*, sequences with a FLAG tag fused to the C‐terminus were cloned into potato virus X vector pGR107 (Jones *et al.*, 1999) by recombinant DNA methods using the GenRec Assembly Master Mix Kit (General Biosystm Co., Ltd) for 127 distinct SCPs, three signal peptide deletion mutants (*VdSCP27* ^ΔSP^, *VdSCP113* ^ΔSP^, *VdSCP126* ^ΔSP^), cysteine site‐directed mutagenesis of *VdSCP27* (C‐A), *VdSCP113* (C‐A), and *VdSCP126* (C‐A, C140A, C172A, C201A, C211A), *VdCBM1*, the BAX protein (used for positive control), and GFP (used for negative control). For functional assays of signal peptides in yeast, the predicted signal peptide encoding sequences of *VdSCP27*, *VdSCP113*, and *VdSCP126* were fused in frame to the secretion‐defective invertase gene in the vector pSUC2 (Jacobs *et al.*, 1997) to form the recombinant constructs (pSUC::SP^VdSCP27^, pSUC::SP^VdSCP113^, and pSUC::SP^VdSCP126^). To study the subcellular localization of VdSCP27, VdSCP113, and VdSCP126 in planta, the full‐length *VdSCP27*, *VdSCP113* *,*and *VdSCP126* cDNAs were introduced into the 5′ end of the gene encoding GFP of the pBinGFP4 vector driven by a cauliflower mosaic virus 35S promoter to generate the recombinant expression vectors (pBin::*VdSCP27‐GFP*, pBin::*VdSCP113‐GFP*, and pBin::*VdSCP126‐GFP*). For VIGS analyses, the genes *BAK1* and *SOBIR1* were amplified from *N. benthamiana* cDNA and integrated into the vector pTRV2 to construct pTRV2::*BAK1* and pTRV2::*SOBIR1*, and the plasmid pTRV2::*GFP* was used as a control. For the targeted gene deletions in *V. dahliae*, the constructs (pGKO::*VdSCP27*, pGKO::*VdSCP113*, and pGKO::*VdSCP126*) were generated based on a previously described method (Liu *et al.*, 2013). Briefly, approximately 1 kb of 5′ and 3′ flanking sequences of the three SCPs were amplified from *V. dahliae* genomic DNA and fused to a 1.8 kb hygromycin‐resistance cassette. The fusion product was introduced into the binary vector pGKO2‐Gateway using a standard BP reaction (the cloned DNA fragment containing the attB site was recombined with the donor vector containing the attP site in vitro to produce the recombinant vector). To construct the double gene deletion mutant in *V. dahliae*, the sequences of *VdSCP27, VdSCP113*, and *VdSCP126* accompanied by two flanking sequences were amplified from *V. dahliae* strain Vd991 genomic DNA and cloned into the binary vector pCOM (pCOM::*VdSCP27*, pCOM::*VdSCP113*, and pCOM::*VdSCP126*) that carries the geneticin resistance gene (Zhou *et al*., 2013), and were subsequently reintroduced to the ∆*VdSCP113*, ∆*VdSCP126*, and ∆*VdSCP27* strains, respectively.

### 4.4 Transient expression assays

Transient gene expression assays were performed in 4‐week‐old *N. benthamiana* plant leaves using the wild type and those strains harbouring mutant alleles including (a) the full‐length coding sequences of 127 *VdSCP*s; (b) *VdSCP27* ^ΔSP^, *VdSCP113* ^ΔSP^, and *VdSCP126* ^ΔSP^, mutants of *VdSCP27*, *VdSCP113* or *VdSCP126* that lacked the sequence encoding the signal peptide; (c) the site‐directed cysteine residue mutants of VdSCP27, VdSCP113, and VdSCP126 (“C‐A” indicates that all cysteine residues were replaced by alanine; “C‐No.‐A” indicates that a single cysteine residue was replaced by an alanine in the respective position in VdSCPs); (d) VdCBM1; and (e) the positive and negative controls BAX and GFP. All constructs were separately transformed into *A. tumefaciens* GV3101 pMP90. Agroinfiltration assays were performed on *N. benthamiana* plants using BAX and GFP as positive and negative controls, respectively. Each assay was performed on six leaves from three individual plants and repeated at least three times. The suppression of cell death induction by VdSCPs was tested by co‐agroinfiltration with appropriate expression constructs encoding *VdSCP27*, *VdSCP113*, and *VdSCP126*. Leaf phenotypes were photographed 6 days after infiltration. Total proteins were extracted using a P‐PER Plant Protein Extraction Kit and Protease Inhibitor Cocktail Kit (Thermo Scientific) from agroinfiltrated *N. benthamiana* leaves 60 hours after infiltration. Transient protein expression in *N. benthamiana* was assessed using anti‐FLAG antibodies (Sigma‐Aldrich).

### 4.5 Yeast signal sequence trap system

Yeast signal sequence trap system functional validation of the predicted signal peptide was performed as described previously (Jacobs *et al.*, 1997). The plasmid constructs pSUC::SP^VdSCP27^, pSUC::SP^VdSCP113^, and pSUC::SP^VdSCP126^ were transformed into the yeast strain YTK12. Positive clones were confirmed by PCR using vector‐specific primers (Table S6). The transformants were grown on CMD–W medium containing sucrose instead of glucose (0.67% yeast N base without amino acids, 0.075% tryptophan dropout supplement, 2% sucrose, 0.1% glucose and 2% agar). To assay invertase secretion, the positive transformants were incubated on YPRAA medium (1% yeast extract, 2% peptone, 2% raffinose, and 2 mg/ml antimycin A) containing raffinose as the carbohydrate source. YTK12 transformed with the functional signal peptide of Avr1b or an empty pSUC2 vector were used as positive and negative controls, respectively (Gu *et al.*, 2011).

### 4.6 Subcellular localization assay

To examine the subcellular localization of three gene products (VdSCP27, VdSCP113, and VdSCP126), the signal peptide‐encoding sequences were deleted in the vectors carrying each of these native genes (pBin::*VdSCP27‐GFP*, pBin::*VdSCP113‐GFP*, and pBin::*VdSCP126‐GFP*) and the resultant constructs of pBin::*VdSCP27* ^∆SP^ *‐GFP*, pBin::*VdSCP113* ^∆SP^ *‐GFP*, and pBin::*VdSCP126* ^∆SP^ *‐GFP*, respectively, were transformed into *A. tumefaciens* GV3101 and agroinfiltrated into 4‐week‐old *N. benthamiana* leaves. The 35S::*GFP* (empty vector of pBinGFP4) was used as a control. To observe fluorescence, the infiltrated tobacco leaves were harvested 2 days post‐agroinfiltration then incubated in the fluorescent dye FM4‐64 for 15 min and imaged under a laser scanning confocal microscope (LSM T‐PMT) with excitation at 488 nm and emission at 510 nm for GFP, and excitation at 543 nm and emission at 562 nm for FM4‐64.

### 4.7 VIGS in *N. benthamiana*

VIGS assays based on recombinant tobacco rattle virus (TRV) were performed as described previously (Liu *et al.*, 2002). The plasmid constructs pTRV1, pTRV2::*BAK1*, pTRV2::*SOBIR1*, and pTRV2::*GFP* were introduced into *A. tumefaciens* GV3101. *Agrobacterium* strains harbouring either the *pTRV2::BAK1* or *pTRV2::SOBIR1* plasmid were combined with strains harbouring the pTRV1 vector in a 1:1 ratio and co‐infiltrated into two primary leaves of 4‐week‐old *N. benthamiana* plants. The plasmid pTRV2::*GFP* was used as the control. The effectiveness of the VIGS assay was evaluated using the phytoene desaturase (*PDS*) gene as previously described (Liu *et al.*, 2002). The silencing efficiencies of *NbBAK1* and *NbSOBIR1* were validated by quantitative reverse transcription PCR (RT‐qPCR) analysis of RNA. The experiment was performed three times with six plants for each TRV construct per experiment.

### 4.8 ROS activity, cellulose deposition, and electrolyte leakage

ROS generation was detected using 3,3′‐diaminobenzidine (DAB) solution (Solarbio) at 2 days after agroinfiltration of the corresponding gene in *N. benthamiana* leaves, as described previously (Bindschedler *et al.*, 2006). Callose deposition was determined under fluorescence microscopy with a UV filter. The *N. benthamiana* leaves were harvested 2 days after agroinfiltration. The infiltrated leaf discs were destained with 75% ethanol at 37 °C and incubated in 150 mM phosphate buffer (pH 9.5) containing aniline blue (approximately 1% wt/vol) (Sigma‐Aldrich) for 2 hr in the dark. The experiments were repeated three times and the relative amounts of ROS and callose deposition were calculated using the software ImageJ. Electrolyte leakage assays were performed as described previously (Oh *et al.*, 2010). Ion conductivity was measured using a conductivity meter with Probe LE703 (Mettler‐Toledo).

### 4.9 Protection against disease development after inoculation with *B. cinerea*

*A. tumefaciens* was transformed with the expression cassettes for *VdSCP27*, *VdSCP113*, and *VdSCP12*6, and the strains harbouring the individual expression cassettes were agroinfiltrated into the whole area of leaves of 4‐week‐old *N. benthamiana* (at a proper concentration to avoid induced cell death)*.* At 12 hr post‐infiltration, 5 μl of 2 × 10^6^ conidia/ml of *B. cinerea* was placed on the infiltrated area. The inoculated plants were placed in an incubator at 25 °C and 80% relative humidity. The diameters of the lesions were measured 48 hr after inoculation. Infection assays were performed using three leaves per plant and experiments were repeated three times.

### 4.10 Gene expression analysis

To determine the expression of *VdSCP27*, *VdSCP113*, and *VdSCP126* during infection, 2‐week‐old cotton and 4‐week‐old *N. benthamiana* seedlings were root‐dip‐inoculated with 5 × 10^6^ conidia/ml *V. dahliae*, and the roots were harvested at different time points. The AxyPreP Multisource Total RNA Miniprep Kit (Axygen) was used to isolate total RNA. RT‐qPCR was performed under the following conditions: an initial 95 °C denaturation step for 10 min, followed by 40 cycles of 95 °C for 15 s and 60 °C for 1 min. The *N. benthamiana* and *V. dahliae EF‐1α* were used as endogenous references. Relative transcript levels among various samples were determined using the 2^−∆∆^ *^C^* ^t^ method from three independent experiments (Livak and Schmittgen, 2001). Unpaired Student's *t* tests were performed to determine statistical significance. The transient expression samples were also used for the expression of defence response genes induced by VdSCP27, VdSCP113, and VdSCP126 or suppressed by VdCBM1 with the same methods. Primers used for expression profiling are listed in Table S6.

### 4.11 Immunoblotting

To verify protein production during transient expression in *N. benthamiana*, total proteins were extracted using the P‐PER Plant Protein Extraction Kit (Thermo Scientific) and Protease Inhibitor Cocktail Kit (Thermo Scientific) from the agroinfiltrated *N. benthamiana* leaves 60 hr after inoculation, following the manufacturer's instructions. The proteins were separated using 15% sodium dodecyl sulphate polyacrylamide electrophoresis gels, and transient protein expression in *N. benthamiana* was assessed using anti‐FLAG M2 antibody (Sigma) and detected using the Pierce ECL western blotting substrate (Thermo Scientific).

### 4.12 *V. dahliae* transformation and virulence assays

The constructs pGKO::*VdSCP27*, pGKO::*VdSCP113*, pGKO::*VdSCP126*, pCOM::*VdSCP27*, pCOM::*VdSCP113*, and pCOM::*VdSCP126* were transferred into *A. tumefaciens* AGL‐1 for fungal transformations. *A. tumefaciens*‐mediated transformation of *V. dahliae* for gene deletion by homologous recombination was conducted as described previously (Mullins and Kang, 2001). The transformants were grown on PDA supplemented with 50 μg/ml hygromycin or 20 μg/ml geneticin for selection of targeted gene deletion mutant strains. Gene replacements in the targeted deletion mutants were verified by PCR using the appropriate primer sets (Table S6). Pathogenicity assays were performed on cotton and *N. benthamiana* seedlings as previously described (Gui *et al.*, 2017) using a root‐dipping method. For fungal biomass quantification, the roots of three plants were harvested at 3 weeks after inoculation and ground to a powder for genomic DNA extraction. The fungal biomass was quantified by quantitative PCR as described by Santhanam *et al. *(2013). *Verticillium* elongation factor 1‐α (*EF‐1α*) was used to quantify fungal colonization, and the cotton *18S rRNA* gene and *N. benthamiana EF‐1α* served as an endogenous plant control. Unpaired Student's *t* tests were performed to determine statistical significance.

## AUTHOR CONTRIBUTIONS

J.Y.C., K.S., and X.F.D. designed the research. D.W. contributed to the identification of the three VdSCPs. J.S. performed the cell death‐inducing activity of all 127 VdSCPs and identified the three that induced cell death. L.T. generated the three VdSCPs deletion mutants and performed the virulence tests. D.D.Z., L.T., L.Z., and J.Y.C. analysed the data. S.S.S., C.M.Y., Y.L., B.L.W., Z.Q.K., J.J.L., J.W., S.A., and T.G.L. assisted with specific experiments. J.Y.C. and D.D.Z. prepared the manuscript, which was edited by S.J.K. and K.S., D.W., L.T., D.D.Z., and J.S. contributed equally. None of the authors have conflicts of interest with this manuscript.

## Data Availability

The data that support the findings of this study are available from the corresponding author upon reasonable request.
