# Supplementary material for: Functional analyses of small secreted cysteine‐rich proteins identified candidate effectors in Verticillium dahliae
Source: Mol Plant Pathol. 2020 Mar 10;21(5):667–85. doi: 10.1111/mpp.12921 (PMC7170778; doi:10.1111/mpp.12921)
Supplement: Supplementary file 1 [file MPP-21-667-s001.doc]

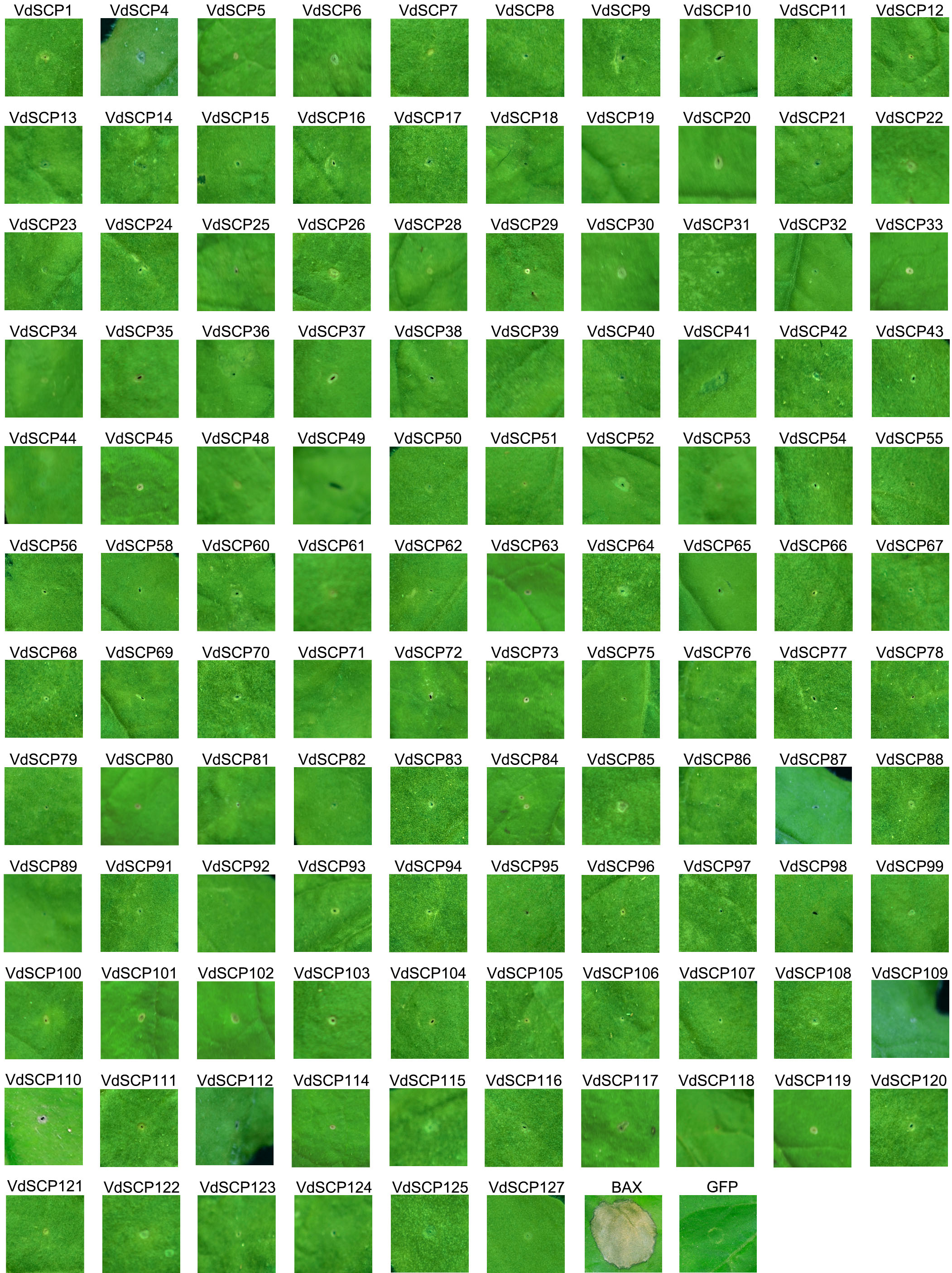


**Figure S1 | Analyses of the cell death-inducing activities of 120 VdSCPs in *Nicotiana benthamiana*.** The non-cell death inducing activity of 120 VdSCPs in *N. benthamiana* leaves from 4-week-old plants 6 days after infiltration with *Agrobacterium tumefaciens* carrying the indicated genes. The BAX (Bcl-2-associated X) and green fluorescent protein (GFP) were used as the positive and negative controls, respectively.
