# Supplementary material for: Functional analyses of small secreted cysteine‐rich proteins identified candidate effectors in Verticillium dahliae
Source: Mol Plant Pathol. 2020 Mar 10;21(5):667–85. doi: 10.1111/mpp.12921 (PMC7170778; doi:10.1111/mpp.12921)
Supplement: Supplementary file 2 [file MPP-21-667-s002.doc]

**
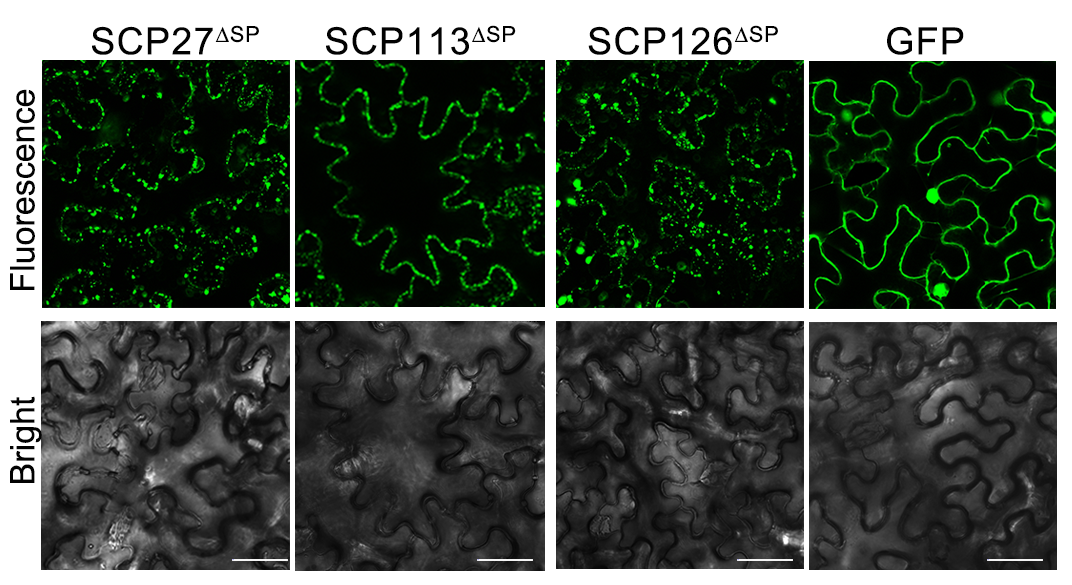
**

**Figure S2 | The subcellular location analysis following signal peptide deletion.** Subcellular localization of VdSCP27*^∆^*^SP^, VdSCP113*^∆^*^SP^ and VdSCP126*^∆^*^SP^ derived from *Verticillium dahliae* were determined by transient expression of C-terminal green fluorescent protein (GFP)-tagged proteins in *N. benthamiana* leaves. The genes were transiently expressed in 4-week-old *N. benthamiana* leaves and harvested at 2 days post-agro-infiltration. The GFP constructs were used as negative controls. Fluorescence was scanned using a Leica TCS SP8 confocal microscopy system using ×200 magnification with an excitation wavelength at 488 nm and emission at 510 nm, and an excitation wavelength at 543 nm and emission at 562 nm for FM4-64. Bars = 25 μm
