# Supplementary material for: Functional analyses of small secreted cysteine‐rich proteins identified candidate effectors in Verticillium dahliae
Source: Mol Plant Pathol. 2020 Mar 10;21(5):667–85. doi: 10.1111/mpp.12921 (PMC7170778; doi:10.1111/mpp.12921)
Supplement: Supplementary file 3 [file MPP-21-667-s003.doc]

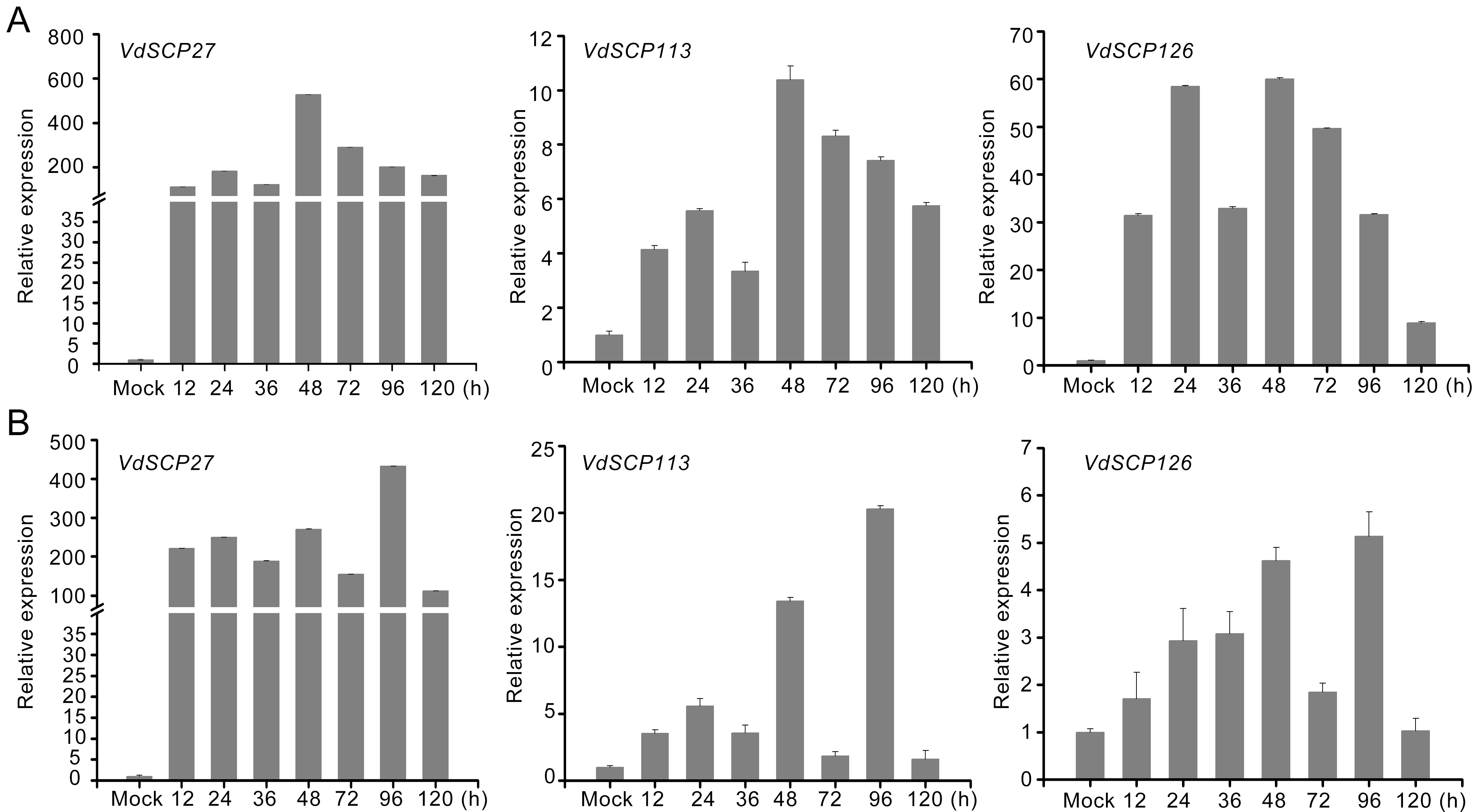


**Figure S3 | The transcript levels of VdSCP27, VdSCP113 and VdSCP126 during infection on host plants.** RT-qPCR analyses were performed for each of the three genes, in both (**A**) *Nicotiana benthamiana* and (**B**) *Gossypium hirsutum*.
