# Supplementary material for: Functional analyses of small secreted cysteine‐rich proteins identified candidate effectors in Verticillium dahliae
Source: Mol Plant Pathol. 2020 Mar 10;21(5):667–85. doi: 10.1111/mpp.12921 (PMC7170778; doi:10.1111/mpp.12921)
Supplement: Supplementary file 4 [file MPP-21-667-s004.doc]

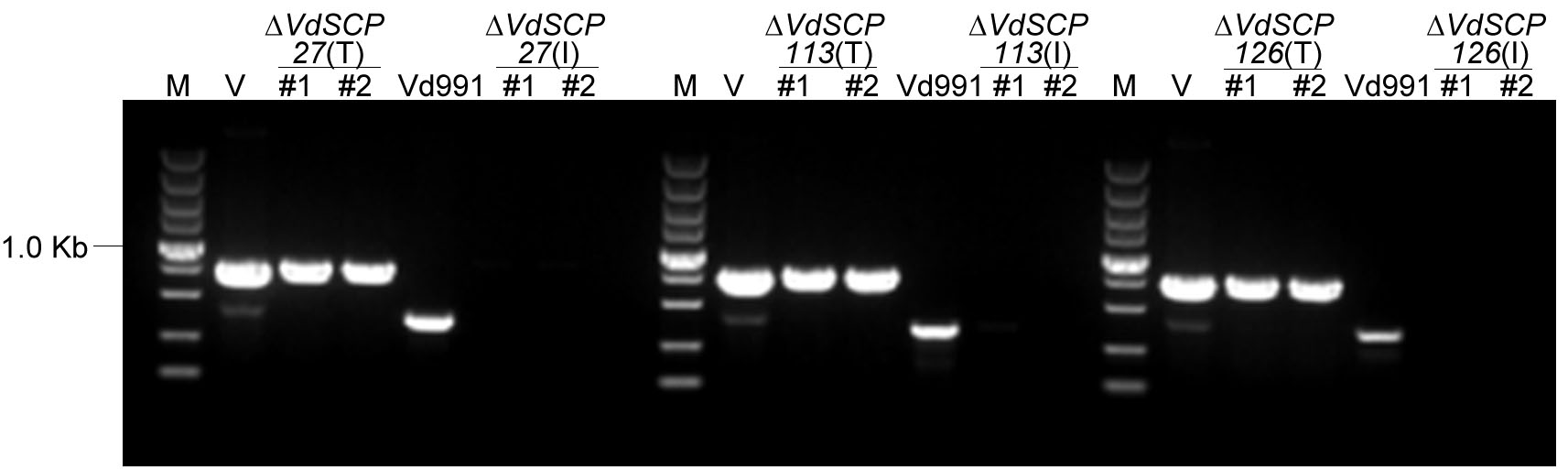


**Figure S4 | Polymerase chain reaction (PCR) analyses of single deletions of *VdSCP27*, *VdSCP113* and *VdSCP126*.** Two transformants (#1 and #2) of each of the deletion mutant strains are shown. *ΔVdSCP27*(T), *ΔVdSCP113*(T) and *ΔVdSCP126*(T): PCR amplification of the positive selection marker hygromycin phosphotransferase; *ΔVdSCP27*(I), *ΔVdSCP113*(I) and *ΔVdSCP126*(I): PCR amplification of markers specific to the internal gene sequence of *VdSCP27*, *VdSCP113* and *VdSCP126*, respectively; Vector pGKO2 (V) and wild-type strain Vd991 were used as the positive controls for hygromycin phosphotransferase and internal gene sequence markers, respectively. M=5,000 bp DNA ladder used as a size marker.
