# Supplementary material for: Functional analyses of small secreted cysteine‐rich proteins identified candidate effectors in Verticillium dahliae
Source: Mol Plant Pathol. 2020 Mar 10;21(5):667–85. doi: 10.1111/mpp.12921 (PMC7170778; doi:10.1111/mpp.12921)
Supplement: Supplementary file 5 [file MPP-21-667-s005.doc]

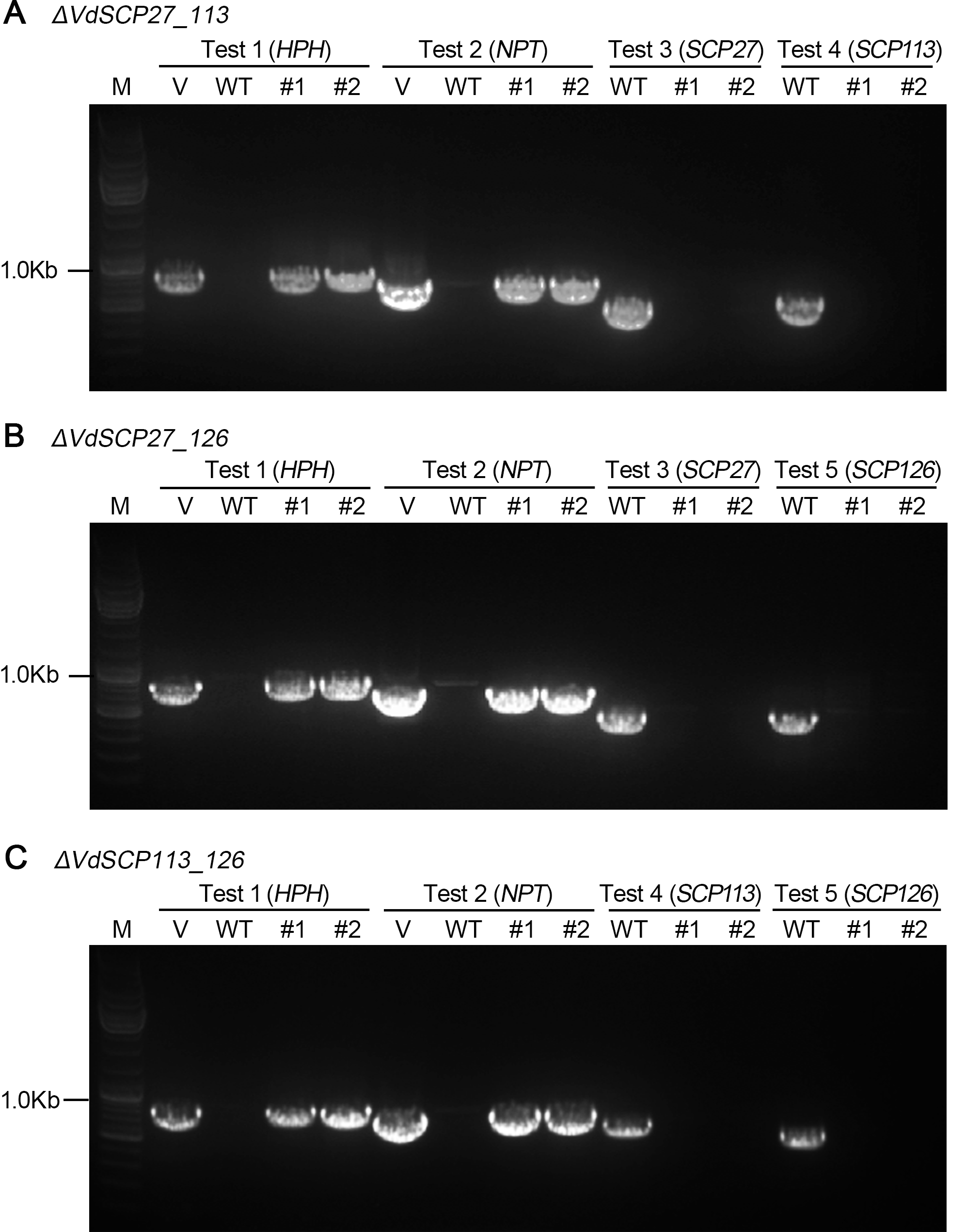


**Figure S5 | Polymerase chain reaction (PCR) analyses of double deletion strains of *VdSCP27*, *VdSCP113*, and *VdSCP126*.** Identification of double deletion mutants Δ*VdSCP27_113* **(A)***,* Δ*VdSCP27_126* **(B)**, and Δ*VdSCP113_126* (**C**). Two transformants (#1 and #2) of each of the deletion mutant strains are shown. Test 1 (HPH): PCR amplification of the positive selection marker hygromycin phosphotransferase; Test 2 (NPT): PCR amplification of the positive selection marker geneticin. Test 3, 4 and 5: PCR amplification of markers specific to the internal gene sequences of *VdSCP27*, *VdSCP113* and *VdSCP126*, respectively; Vector pGKO2 (Test 1 V), Vector Pcom (Test 2 V) and wild-type strain Vd991 were used as the positive controls for hygromycin phosphotransferase, geneticin and internal gene sequence markers, respectively. M=5,000 bp DNA ladder used as a size marker.
