# Supplementary material for: Functional analyses of small secreted cysteine‐rich proteins identified candidate effectors in Verticillium dahliae
Source: Mol Plant Pathol. 2020 Mar 10;21(5):667–85. doi: 10.1111/mpp.12921 (PMC7170778; doi:10.1111/mpp.12921)
Supplement: Supplementary file 6 [file MPP-21-667-s006.doc]

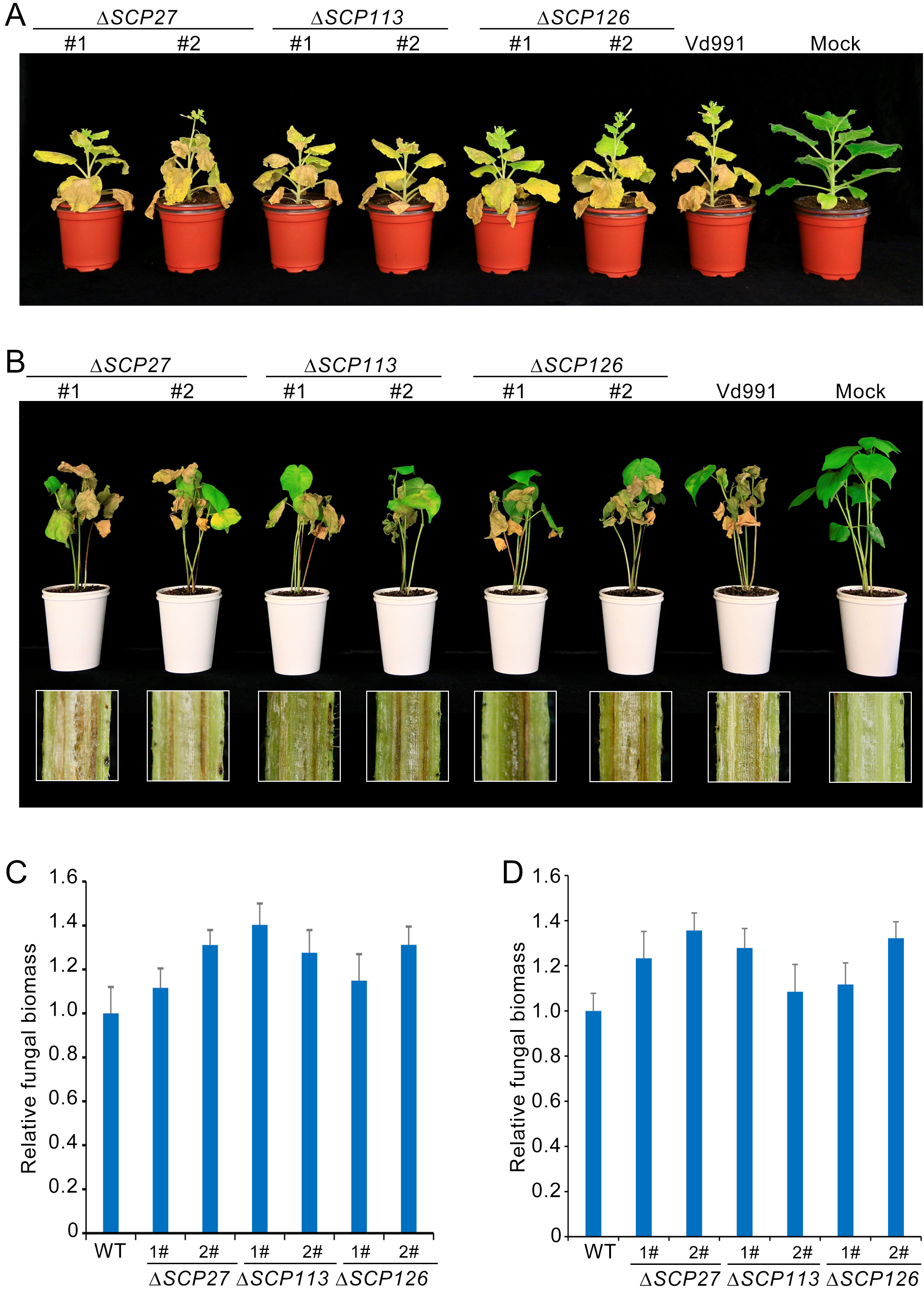


**Figure S6 | Analyses of virulence of the *VdSCP27*, *VdSCP113,* and *VdSCP126* deletion strains in *Nicotina benthamiana* and *Gossypium hirsutum* interactions.** **(A)** Phenotypes of *N. benthamiana* plants inoculated with the targeted gene deletion mutants of *VdSCP27*, *VdSCP113*, and *VdSCP126*. 4-week-old seedlings of *N. benthamiana* plants were inoculated with respective *VdSCPs* gene deletion strains (∆*VdSCP27,* ∆*VdSCP113* and ∆*VdSCP126*, two independent transformations for each gene), wild-type *V. dahliae* (Vd991), and sterile water (Mock). Verticillium wilt symptoms were photographed three weeks after inoculation. (**B**) Pathogenicity assay to investigate the role of VdSCP27, VdSCP113, and VdSCP126 in *V. dahliae* virulence on *Gossypium hirsutum* cv. Junmian No. 1. Quantitative PCR analyses of *in planta* fungal biomass for the *VdSCP27*, *VdSCP113*, and *VdSCP126* deletion strains that were inoculated onto (**C**) *N. benthamiana* and (**D**) *Gossypium hirsutum*. Error bars represent standard errors.
