# Supplementary material for: Functional analyses of small secreted cysteine‐rich proteins identified candidate effectors in Verticillium dahliae
Source: Mol Plant Pathol. 2020 Mar 10;21(5):667–85. doi: 10.1111/mpp.12921 (PMC7170778; doi:10.1111/mpp.12921)
Supplement: Supplementary file 8 [file MPP-21-667-s008.docx]

| **Table S2 Analyses of the position of cysteine residues in VdSCPs** | |
| --- | --- |
| **Alias name** | **Cys position in SCPs** |
| VdSCP1 | Cys52-Cys81-Cys118-Cys133 |
| VdSCP2 | Cys29-Cys45-Cys47-Cys59-Cys61-Cys74-Cys83-Cys100-Cys102-Cys110-Cys112-Cys125 |
| VdSCP3 | Cys17-Cys54-Cys63-Cys259 |
| VdSCP4 | Cys7-Cys27-Cys45-Cys47-Cys52 |
| VdSCP5 | Cys84-Cys140-Cys207-Cys222 |
| VdSCP6 | Cys25-Cys71-Cys82-Cys96-Cys111-Cys130-Cys140-Cys144-Cys156-Cys172-Cys202-Cys257-Cys325-Cys361 |
| VdSCP7 | Cys39-Cys44-Cys58-Cys63-Cys68-Cys221-Cys233-Cys282-Cys285 |
| VdSCP8 | Cys87-Cys128-Cys133-Cys142-Cys165-Cys168-Cys177-Cys196 |
| VdSCP9 | Cys15-Cys44-Cys52-Cys73-Cys75 |
| VdSCP10 | Cys7-Cys13-Cys42-Cys49-Cys66-Cys93-Cys214-Cys236 |
| VdSCP11 | Cys96-Cys115-Cys117-Cys148-Cys151 |
| VdSCP12 | Cys31-Cys58-Cys114-Cys117-Cys127-Cys143-Cys151-Cys153-Cys156-Cys169-Cys246-Cys252-Cys255-Cys257-Cys259-Cys263-Cys268-Cys272-Cys274 |
| VdSCP13 | Cys55-Cys75-Cys83-Cys108-Cys210-Cys261 |
| VdSCP14 | Cys45-Cys51-Cys55-Cys57-Cys67-Cys81-Cys136-Cys142-Cys146-Cys148-Cys153-Cys167 |
| VdSCP15 | Cys43-Cys77-Cys114-Cys127 |
| VdSCP16 | Cys115-Cys137-Cys144-Cys154-Cys164-Cys231 |
| VdSCP17 | Cys14-Cys61-Cys69-Cys102-Cys112-Cys215 |
| VdSCP18 | Cys54-Cys63-Cys98-Cys109 |
| VdSCP19 | Cys43-Cys138-Cys191-Cys233-Cys258 |
| VdSCP20 | Cys22-Cys29-Cys31-Cys64-Cys80-Cys91-Cys129-Cys154-Cys168-Cys202-Cys262-Cys271-Cys280-Cys343-Cys391 |
| VdSCP21 | Cys22-Cys31-Cys42-Cys49-Cys51-Cys60-Cys64-Cys81 |
| VdSCP22 | Cys30-Cys38-Cys51-Cys62-Cys79-Cys91-Cys95-Cys113-Cys138 |
| VdSCP23 | Cys41-Cys57-Cys68-Cys84-Cys86-Cys97-Cys100-Cys127 |
| VdSCP24 | Cys31-Cys38-Cys51-Cys63-Cys69-Cys80 |
| VdSCP25 | Cys40-Cys53-Cys63-Cys79-Cys81-Cys92-Cys95-Cys122 |
| VdSCP26 | Cys70-Cys84-Cys95-Cys125-Cys135 |
| VdSCP27 | Cys46-Cys86-Cys169-Cys214 |
| VdSCP28 | Cys14-Cys39-Cys121-Cys135-Cys175-Cys188-Cys212-Cys231 |
| VdSCP29 | Cys21-Cys39-Cys47-Cys75 |
| VdSCP30 | Cys61-Cys68-Cys87-Cys124-Cys131-Cys150-Cys184-Cys191-Cys209 |
| VdSCP31 | Cys40-Cys59-Cys80-Cys108-Cys111-Cys118-Cys172-Cys180-Cys209-Cys217 |
| VdSCP32 | Cys39-Cys82-Cys141-Cys159 |
| **Alias name** | **Cys position in SCRs** |
| VdSCP33 | Cys24-Cys28-Cys38-Cys45-Cys47-Cys62-Cys67-Cys83 |
| VdSCP34 | Cys37-Cys69-Cys74-Cys159-Cys165-Cys183-Cys264 |
| VdSCP35 | Cys32-Cys51-Cys73-Cys77-Cys81-Cys94-Cys100-Cys115-Cys134-Cys152 |
| VdSCP36 | Cys8-Cys77-Cys123-Cys136-Cys160-Cys271-Cys280-Cys328-Cys333 |
| VdSCP37 | Cys47-Cys122-Cys157-Cys178 |
| VdSCP38 | Cys56-Cys169-Cys183-Cys229 |
| VdSCP39 | Cys51-Cys55-Cys71-Cys80-Cys85-Cys103-Cys109-Cys139-Cys244 |
| VdSCP40 | Cys41-Cys51-Cys68-Cys79 |
| VdSCP41 | Cys29-Cys33-Cys43-Cys50-Cys52-Cys65-Cys71-Cys87 |
| VdSCP42 | Cys28-Cys57-Cys62-Cys76 |
| VdSCP43 | Cys93-Cys97-Cys107-Cys114-Cys116-Cys129-Cys136-Cys152 |
| VdSCP44 | Cys11-Cys94-Cys106-Cys155-Cys296-Cys314 |
| VdSCP45 | Cys47-Cys51-Cys64-Cys76-Cys79-Cys100-Cys260 |
| VdSCP46 | Cys17-Cys19-Cys47-Cys78 |
| VdSCP47 | Cys25-Cys47-Cys69-Cys78-Cys83-Cys329 |
| VdSCP48 | Cys87-Cys92-Cys94-Cys104-Cys107-Cys129-Cys135-Cys137-Cys145-Cys152 |
| VdSCP49 | Cys48-Cys58-Cys179-Cys202 |
| VdSCP50 | Cys28-Cys35-Cys45-Cys47-Cys52-Cys64-Cys82-Cys90-Cys102-Cys110-Cys122-Cys130-Cys142-Cys150 |
| VdSCP51 | Cys31-Cys36-Cys54-Cys146-Cys156-Cys158-Cys315-Cys326-Cys337-Cys357 |
| VdSCP52 | Cys21-Cys32-Cys36-Cys43-Cys50-Cys54-Cys65-Cys90-Cys96-Cys103-Cys110-Cys114-Cys125-Cys149-Cys154-Cys161-Cys168-Cys172-Cys183-Cys192 |
| VdSCP53 | Cys43-Cys81-Cys119-Cys138-Cys192 |
| VdSCP54 | Cys28-Cys35-Cys62-Cys69-Cys99-Cys106-Cys136-Cys143-Cys173-Cys180-Cys196 |
| VdSCP55 | Cys23-Cys28-Cys49-Cys100-Cys109-Cys111-Cys135 |
| VdSCP56 | Cys37-Cys42-Cys48-Cys57-Cys63-Cys68-Cys74-Cys76-Cys80-Cys83-Cys90-Cys95 |
| VdSCP57 | Cys10-Cys79-Cys186-Cys213 |
| VdSCP58 | Cys48-Cys64-Cys68-Cys128-Cys153-Cys176-Cys184-Cys196-Cys259 |
| VdSCP59 | Cys13-Cys69-Cys120-Cys126 |
| VdSCP60 | Cys30-Cys64-Cys74-Cys82-Cys108-Cys111-Cys128-Cys155-Cys165 |
| VdSCP61 | Cys22-Cys85-Cys207-Cys317-Cys321 |
| VdSCP62 | Cys77-Cys140-Cys185-Cys195 |
| VdSCP63 | Cys192-Cys231-Cys237-Cys333-Cys335-Cys339 |
| VdSCP64 | Cys66-Cys142-Cys162-Cys175-Cys201-Cys237-Cys242-Cys284 |
| VdSCP65 | Cys34-Cys40-Cys48-Cys52-Cys65-Cys72-Cys74-Cys76 |
| **Alias name** | **Cys position in SCRs** |
| VdSCP66 | Cys15-Cys118-Cys144-Cys171-Cys224-Cys254-Cys258-Cys287-Cys298-Cys300-Cys312 |
| VdSCP67 | Cys20-Cys32-Cys110-Cys131-Cys139-Cys155-Cys164-Cys183-Cys247 |
| VdSCP68 | Cys9-Cys40-Cys44-Cys49-Cys51-Cys57-Cys69 |
| VdSCP69 | Cys17-Cys45-Cys47-Cys58-Cys68-Cys75-Cys81-Cys89-Cys101 |
| VdSCP70 | Cys83-Cys97-Cys104-Cys127-Cys138-Cys148-Cys181-Cys191-Cys205-Cys211 |
| VdSCP71 | Cys55-Cys168-Cys182-Cys228 |
| VdSCP72 | Cys25-Cys29-Cys40-Cys47-Cys49-Cys64-Cys69-Cys86 |
| VdSCP73 | Cys83-Cys85-Cys90-Cys110-Cys117-Cys134-Cys136-Cys151-Cys153-Cys157-Cys168-Cys172-Cys176 |
| VdSCP74 | Cys17-Cys40-Cys72-Cys74-Cys93-Cys131-Cys195 |
| VdSCP75 | Cys80-Cys174-Cys204-Cys243-Cys251 |
| VdSCP76 | Cys22-Cys26-Cys39-Cys47-Cys49-Cys61-Cys66-Cys81 |
| VdSCP77 | Cys26-Cys30-Cys43-Cys51-Cys53-Cys65-Cys67-Cys70-Cys87-Cys101 |
| VdSCP78 | Cys76-Cys104-Cys124-Cys128 |
| VdSCP79 | Cys18-Cys23-Cys27-Cys34-Cys36-Cys48-Cys54-Cys60-Cys77-Cys84-Cys88-Cys95-Cys97-Cys104-Cys110-Cys117 |
| VdSCP80 | Cys46-Cys78-Cys115-Cys128 |
| VdSCP81 | Cys54-Cys96-Cys134-Cys152 |
| VdSCP82 | Cys44-Cys51-Cys79-Cys84-Cys196-Cys225 |
| VdSCP83 | Cys46-Cys52-Cys57-Cys62-Cys68-Cys77 |
| VdSCP84 | Cys21-Cys93-Cys198-Cys265-Cys292-Cys338-Cys355 |
| VdSCP85 | Cys13-Cys125-Cys141-Cys172-Cys179-Cys196-Cys225-Cys235-Cys267-Cys270-Cys304 |
| VdSCP86 | Cys23-Cys30-Cys47-Cys58-Cys90-Cys100-Cys105-Cys132-Cys308-Cys357 |
| VdSCP87 | Cys30-Cys47-Cys79-Cys104 |
| VdSCP88 | Cys23-Cys34-Cys40-Cys49-Cys53-Cys59-Cys67-Cys75-Cys83-Cys91-Cys100-Cys104-Cys123-Cys131-Cys137-Cys145-Cys154-Cys158 |
| VdSCP89 | Cys16-Cys36-Cys38-Cys57-Cys149-Cys171-Cys177-Cys220 |
| VdSCP90 | Cys92-Cys118-Cys146-Cys148-Cys190-Cys192-Cys252-Cys277 |
| VdSCP91 | Cys28-Cys61-Cys63-Cys79-Cys82-Cys105-Cys109 |
| VdSCP92 | Cys99-Cys209-Cys242-Cys254 |
| VdSCP93 | Cys48-Cys64-Cys68-Cys128-Cys151-Cys173-Cys181-Cys193 |
| VdSCP94 | Cys26-Cys57-Cys91-Cys99-Cys108-Cys119-Cys122 |
| VdSCP95 | Cys5-Cys256-Cys270-Cys288-Cys335-Cys363 |
| VdSCP96 | Cys47-Cys106-Cys119-Cys350 |
| VdSCP97 | Cys27-Cys146-Cys213-Cys231-Cys236-Cys244 |
| VdSCP98 | Cys52-Cys59-Cys119-Cys123-Cys241-Cys306 |
| **Alias name** | **Cys position in SCRs** |
| VdSCP99 | Cys30-Cys34-Cys45-Cys52-Cys54-Cys66-Cys71-Cys87-Cys95-Cys101 |
| VdSCP100 | Cys46-Cys112-Cys147-Cys164 |
| VdSCP101 | Cys62-Cys106-Cys248-Cys303-Cys348-Cys355-Cys365 |
| VdSCP102 | Cys173-Cys224-Cys240-Cys244-Cys291 |
| VdSCP103 | Cys10-Cys33-Cys80-Cys85-Cys104-Cys106-Cys129-Cys133-Cys161-Cys171 |
| VdSCP104 | Cys37-Cys44-Cys52-Cys54-Cys66-Cys69-Cys90-Cys123 |
| VdSCP105 | Cys49-Cys154-Cys190-Cys199-Cys221 |
| VdSCP106 | Cys74-Cys154-Cys159-Cys163-Cys184-Cys188-Cys278 |
| VdSCP107 | Cys10-Cys123-Cys172-Cys187-Cys195 |
| VdSCP108 | Cys47-Cys53-Cys90-Cys96-Cys128-Cys134-Cys171-Cys177-Cys213-Cys219 |
| VdSCP109 | Cys93-Cys97-Cys106-Cys113-Cys115-Cys123-Cys130-Cys134-Cys136-Cys138 |
| VdSCP110 | Cys72-Cys80-Cys88-Cys105-Cys156 |
| VdSCP111 | Cys47-Cys51-Cys60-Cys64-Cys84-Cys89-Cys163-Cys173-Cys203-Cys207-Cys220-Cys223 |
| VdSCP112 | Cys19-Cys23-Cys44-Cys92-Cys102-Cys104-Cys129-Cys322 |
| VdSCP113 | Cys30-Cys36-Cys57-Cys123-Cys133-Cys135-Cys159 |
| VdSCP114 | Cys120-Cys173-Cys188-Cys196 |
| VdSCP115 | Cys9-Cys33-Cys38-Cys50-Cys70-Cys90-Cys93-Cys108-Cys110-Cys139 |
| VdSCP116 | Cys26-Cys30-Cys43-Cys50-Cys52-Cys63-Cys65-Cys70-Cys86-Cys100 |
| VdSCP117 | Cys6-Cys38-Cys78-Cys98-Cys119-Cys140-Cys159 |
| VdSCP118 | Cys54-Cys75-Cys110-Cys156 |
| VdSCP119 | Cys29-Cys33-Cys43-Cys241-Cys243-Cys257-Cys260-Cys278 |
| VdSCP120 | Cys28-Cys32-Cys43-Cys50-Cys52-Cys67-Cys72-Cys89 |
| VdSCP121 | Cys15-Cys245-Cys254-Cys287-Cys310-Cys378-Cys381 |
| VdSCP122 | Cys28-Cys78-Cys97-Cys133-Cys155-Cys178 |
| VdSCP123 | Cys39-Cys60-Cys152-Cys167-Cys175-Cys198 |
| VdSCP124 | Cys25-Cys29-Cys56-Cys62-Cys104-Cys113-Cys132-Cys134-Cys158-Cys164 |
| VdSCP125 | Cys38-Cys42-Cys51-Cys57-Cys59-Cys70-Cys72-Cys77-Cys93-Cys110 |
| VdSCP126 | Cys140-Cys172-Cys201-Cys211 |
| VdSCP127 | Cys39-Cys49-Cys82-Cys142-Cys163-Cys167 |

Note: Cys, Cysteine; SCRs, small Cysteine rich proteins. VdSCPs in gray color were excluded from small cysteine rich proteins.
