# Supplementary material for: Functional analyses of small secreted cysteine‐rich proteins identified candidate effectors in Verticillium dahliae
Source: Mol Plant Pathol. 2020 Mar 10;21(5):667–85. doi: 10.1111/mpp.12921 (PMC7170778; doi:10.1111/mpp.12921)
Supplement: Supplementary file 10 [file MPP-21-667-s010.docx]

**Table S4 Analysis the distribution pattern of cysteine residues in VdSCPs**

| **Alias name** | **Cys distribution pattern** | **Cys pattern group** |
| --- | --- | --- |
| VdSCP33 | X7-C-X3-C-X9-C-X6-C-X1-C-X14-C-X4-C-X15-C-X93 | **A** |
| VdSCP41 | X11-C-X3-C-X9-C-X6-C-X1-C-X12-C-X5-C-X15-C-X120 | **A** |
| VdSCP43 | X73-C-X3-C-X9-C-X6-C-X1-C-X12-C-X6-C-X15-C-X61 | **A** |
| VdSCP72 | X7-C-X3-C-X10-C-X6-C-X1-C-X14-C-X4-C-X16-C-X110 | **A** |
| VdSCP76 | X2-C-X3-C-X12-C-X7-C-X1-C-X11-C-X4-C-X14-C-X163 | **A** |
| VdSCP77 | X3-C-X3-C-X12-C-X7-C-X1-C-X11-C-X1-C-X2-C-X16-C-X13-C-X1 | **A** |
| VdSCP99 | X7-C-X3-C-X10-C-X6-C-X1-C-X11-C-X4-C-X15-C-X7-C-X5-C | **A** |
| VdSCP116 | X7-C-X3-C-X12-C-X6-C-X1-C-X10-C-C-X4-C-X15-C-X13-C-X67 | **A** |
| VdSCP120 | X10-C-X3-C-X10-C-X6-C-X1-C-X14-C-X4-C-X16-C-X85 | **A** |
| VdSCP125 | X15-C-X3-C-X8-C-X5-C-X1-C-X10-C-C-X4-C-X15-C-X16-C-X113 | **A** |
| VdSCP58 | X24-C-X15-C-X3-C-X59-C-X24-C-X22-C-X7-C-X11-C-X62-C-X4 | **B** |
| VdSCP93 | X28-C-X15-C-X3-C-X59-C-X22-C-X21-C-X7-C-X11-C-X29 | **B** |
| VdSCP107 | X102-C-X48-C-X14-C-X7-C-X6 | **C** |
| VdSCP114 | X99-C-X52-C-X14-C-X7-C-X6 | **C** |
| VdSCP23 | X18-C-X15-C-X10-C-X15-C-X1-C-X10-C-X2-C-X26-C-X13 | **D** |
| VdSCP25 | X19-C-X12-C-X9-C-X15-C-X1-C-X10-C-X2-C-X26-C-X138 | **D** |
| VdSCP55 | X6-C-X4-C-X20-C-X50-C-X8-C-X1-C-X23-C-X3 | **E** |
| VdSCP112 | X1-C-X3-C-X20-C-X47-C-X9-C-X1-C-X24-C-X192-C-X20 | **E** |
| VdSCP113 | X10-C-X5-C-X20-C-X65-C-X9-C-X1-C-X23-C-X32 | **E** |
| VdSCP38 | X36-C-X112-C-X13-C-X45-C-X68 | **F** |
| VdSCP71 | X35-C-X112-C-X13-C-X45-C-X83 | **F** |
| VdSCP1 | X32-C-X28-C-X36-C-X14-C-X59 |  |
| VdSCP2 | X8-C-X15-C-C-X11-C-C-X12-C-X8-C-X16-C-C-X7-C-C-X12-C-X8 |  |
| VdSCP3 | X33-C-X8-C-X195-C-X3 |  |
| VdSCP4 | X3-C-X17-C-C-X4-C-X9 |  |
| VdSCP5 | X66-C-X55-C-X66-C-X14-C-X2 |  |
| **Alias name** | **Cys distribution pattern** | **Cys pattern group** |
| VdSCP6 | X6-C-X45-C-X10-C-X13-C-X14-C-X18-C-X9-C-X3-C-X11-C-X15-C-X29-C-X54-C-X67-C-X35 |  |
| VdSCP7 | X18-C-X4-C-X13-C-X4-C-X4-C-X152-C-X11-C-X48-C-X2-C-X69 |  |
| VdSCP8 | X67-C-X40-C-X4-C-X8-C-X22-C-X2-C-X8-C-X18-C |  |
| VdSCP9 | X22-C-X7-C-X20-C-X1-C-X78 |  |
| VdSCP10 | X23-C-X6-C-X16-C-X26-C-X120-C-X21-C-X57 |  |
| VdSCP11 | X73-C-X18-C-X1-C-X30-C-X2-C-X60 |  |
| VdSCP12 | X10-C-X26-C-X55-C-X2-C-X9-C-X15-C-X7-C-X1-C-X2-C-X12-C-X76-C-X5-C-X2-C-C-C-X3-C-X4-C-X3-C-X1 |  |
| VdSCP13 | X33-C-X19-C-X7-C-X24-C-X101-C-X50-C-X3 |  |
| VdSCP14 | X26-C-X5-C-X3-C-C-X9-C-X13-C-X54-C-X5-C-X3-C-C-X4-C-X13-C-X5 |  |
| VdSCP15 | X24-C-X33-C-X36-C-X12-C-X87 |  |
| VdSCP16 | X91-C-X21-C-X6-C-X9-C-X9-C-X66-C-X71 |  |
| VdSCP17 | X37-C-X7-C-X32-C-X9-C-X102-C-X8 |  |
| VdSCP18 | X14-C-X8-C-X34-C-X10-C-X123 |  |
| VdSCP19 | X19-C-X94-C-X52-C-X41-C-X24-C-X58 |  |
| VdSCP20 | X3-C-C-X32-C-X15-C-X10-C-X37-C-X24-C-X13-C-X33-C-X59-C-X8-C-X8-C-X62-C-X47-C-X7 |  |
| VdSCP21 | X3-C-X8-C-X10-C-X6-C-X1-C-X8-C-X3-C-X16-C-X86 |  |
| VdSCP22 | X11-C-X7-C-X12-C-X10-C-X16-C-X11-C-X3-C-X17-C-X24-C-X102 |  |
| VdSCP24 | X10-C-X6-C-X12-C-X11-C-X5-C-X10-C-X1 |  |
| VdSCP26 | X50-C-X13-C-X10-C-X29-C-X9-C-X20 |  |
| VdSCP27 | X28-C-X39-C-X82-C-X44-C |  |
| VdSCP28 | X14-C-X81-C-X13-C-X39-C-X12-C-X23-C-X18-C-X13 |  |
| VdSCP29 | X4-C-X17-C-X7-C-X27-C-X93 |  |
| VdSCP30 | X39-C-X6-C-X18-C-X36-C-X6-C-X18-C-X33-C-X6-C-X17-C-X82 |  |
| VdSCP31 | X12-C-X18-C-X20-C-X27-C-X2-C-X6-C-X53-C-X7-C-X28-C-X7-C-X1 |  |
| VdSCP32 | X18-C-X42-C-X58-C-X17-C-X5 |  |
| VdSCP34 | X18-C-X31-C-X4-C-X84-C-X5-C-X17-C-X80-C-X26 |  |
| VdSCP35 | X12-C-X18-C-X21-C-X3-C-X3-C-X12-C-X5-C-X14-C-X18-C-X17-C-X1 |  |
| VdSCP36 | X60-C-X45-C-X12-C-X23-C-X110-C-X8-C-X47-C-X4-C-X19 |  |
| **Alias name** | **Cys distribution pattern** | **Cys pattern group** |
| VdSCP37 | X29-C-X74-C-X34-C-X20-C-X53 |  |
| VdSCP39 | X30-C-X3-C-X15-C-X8-C-X4-C-X17-C-X5-C-X29-C-X104-C-X11 |  |
| VdSCP40 | X24-C-X9-C-X16-C-X10-C-X11 |  |
| VdSCP42 | X5-C-X28-C-X4-C-X13-C-X142 |  |
| VdSCP44 | X73-C-X11-C-X48-C-X140-C-X17-C-X35 |  |
| VdSCP45 | X25-C-X3-C-X12-C-X11-C-X2-C-X20-C-X159-C-X105 |  |
| VdSCP46 | X12-C-X30-C-X31 |  |
| VdSCP47 | X3-C-X21-C-X21-C-X8-C-X4-C-X245-C-X1 |  |
| VdSCP48 | X64-C-X4-C-X1-C-X9-C-X2-C-X21-C-X5-C-C-X7-C-X6-C-X4 |  |
| VdSCP49 | X28-C-X9-C-X120-C-X22-C-X22 |  |
| VdSCP50 | X8-C-X6-C-X9-C-X1-C-X4-C-X11-C-X17-C-X7-C-X11-C-X7-C-X11-C-X7-C-X11-C-X7-C-X46 |  |
| VdSCP51 | X8-C-X4-C-X17-C-X91-C-X9-C-X1-C-X156-C-X10-C-X10-C-X19-C-X10 |  |
| VdSCP52 | X3-C-X10-C-X3-C-X6-C-X6-C-X3-C-X10-C-X24-C-X5-C-X6-C-X6-C-X3-C-X10-C-X23-C-X4-C-X6-C-X6-C-X3-C-X10-C-X8 |  |
| VdSCP53 | X23-C-X37-C-X37-C-X18-C-X53-C-X8 |  |
| VdSCP54 | X8-C-X6-C-X26-C-X6-C-X29-C-X6-C-X29-C-X6-C-X29-C-X6-C-X15-C-X16 |  |
| VdSCP56 | X10-C-X4-C-X5-C-X8-C-X5-C-X4-C-X5-C-C-X3-C-X2-C-X6-C-X4-C-X6 |  |
| VdSCP57 | X57-C-X106-C-X26-C-X153 |  |
| VdSCP59 | X48-C-X50-C-X5-C-X99 |  |
| VdSCP60 | X5-C-X33-C-X9-C-X7-C-X25-C |  |
| VdSCP61 | X1-C-X62-C-X121-C-X109-C-X3-C-X62 |  |
| VdSCP62 | X56-C-X62-C-X44-C-X9-C-X40 |  |
| VdSCP63 | X172-C-X38-C-X5-C-X95-C-X1-C-X3-C-X33 |  |
| VdSCP64 | X48-C-X75-C-X19-C-X12-C-X25-C-X35-C-X4-C-X41-C-X49 |  |
| VdSCP65 | X15-C-X5-C-X7-C-X3-C-X12-C-X6-C-C-C-X3 |  |
| VdSCP66 | X98-C-X25-C-X26-C-X52-C-X29-C-X3-C-X28-C-X10-C-C-X11-C-X29 |  |
| VdSCP67 | X7-C-X77-C-X20-C-X7-C-X15-C-X8-C-X18-C-X63-C-X44 |  |
| VdSCP68 | X17-C-X3-C-X4-C-C-X5-C-X11-C-X203 |  |
| VdSCP69 | X25-C-C-X10-C-X9-C-X6-C-X5-C-X7-C-X11-C-X25 |  |
| **Alias name** | **Cys distribution pattern** | **Cys pattern group** |
| VdSCP70 | X66-C-X13-C-X6-C-X22-C-X10-C-X9-C-X32-C-X9-C-X13-C-X5-C-X9 |  |
| VdSCP73 | X61-C-X1-C-X4-C-X19-C-X6-C-X16-C-X1-C-X14-C-X1-C-X3-C-X10-C-X3-C-X3-C-X92 |  |
| VdSCP74 | X21-C-X31-C-X1-C-X18-C-X37-C-X63-C-X3 |  |
| VdSCP75 | X59-C-X93-C-X29-C-X38-C-X7-C-X26 |  |
| VdSCP78 | X53-C-X27-C-X19-C-X3-C-X23 |  |
| VdSCP79 | X-C-X4-C-X3-C-X6-C-C-X11-C-X5-C-X5-C-X16-C-X6-C-X3-C-X6-C-C-X6-C-X5-C-X6-C-X157 |  |
| VdSCP80 | X26-C-X31-C-X36-C-X12-C-X90 |  |
| VdSCP81 | X31-C-X41-C-X37-C-X17-C-X90 |  |
| VdSCP82 | X27-C-X6-C-X27-C-X4-C-X111-C-X28-C-X4 |  |
| VdSCP83 | X27-C-X5-C-X4-C-X4-C-X5-C-X8-C |  |
| VdSCP84 | X-C-X71-C-X104-C-X66-C-X26-C-X45-C-X16-C |  |
| VdSCP85 | X103-C-X15-C-X30-C-X6-C-X16-C-X28-C-X9-C-X31-C-X2-C-X33-C-X41 |  |
| VdSCP86 | X2-C-X6-C-X16-C-X10-C-X31-C-X9-C-X4-C-X26-C-X175-C-X48-C-X2 |  |
| VdSCP87 | X9-C-X16-C-X31-C-X24-C-X15 |  |
| VdSCP88 | X3-C-X10-C-X5-C-X8-C-X3-C-X5-C-X7-C-X7-C-X7-C-X7-C-X8-C-X3-C-X18-C-X7-C-X5-C-X7-C-X8-C-X3-C-X5 |  |
| VdSCP89 | X11-C-X1-C-X18-C-X91-C-X21-C-X5-C-X42-C-X95 |  |
| VdSCP90 | X71-C-X25-C-X27-C-X1-C-X41-C-X1-C-X59-C-X24-C-X18 |  |
| VdSCP91 | X6-C-X32-C-X1-C-X15-C-X2-C-X22-C-X3-C-X8 |  |
| VdSCP92 | X80-C-X109-C-X32-C-X11-C-X7 |  |
| VdSCP94 | X9-C-X30-C-X33-C-X7-C-X8-C-X10-C-X2-C-X147 |  |
| VdSCP95 | X236-C-X13-C-X17-C-X46-C-X27-C-X24 |  |
| VdSCP96 | X26-C-X58-C-X12-C-X230-C-X9 |  |
| VdSCP97 | X7-C-X118-C-X66-C-X17-C-X4-C-X7-C-X63 |  |
| VdSCP98 | X26-C-X6-C-X59-C-X3-C-X117-C-X64-C-X74 |  |
| VdSCP100 | X27-C-X65-C-X34-C-X16-C-X74 |  |
| VdSCP101 | X41-C-X43-C-X141-C-X54-C-X44-C-X6-C-X9-C-X27 |  |
| VdSCP102 | X152-C-X50-C-X15-C-X3-C-X46-C-X23- |  |
| VdSCP103 | X15-C-X46-C-X4-C-X18-C-X1-C-X22-C-X3-C-X27-C-X9-C-X8- |  |
| **Alias name** | **Cys distribution pattern** | **Cys pattern group** |
| VdSCP104 | X10-C-X6-C-X7-C-C-X11-C-X2-C-X20-C-X32-C-X59 |  |
| VdSCP105 | X31-C-X104-C-X35-C-X8-C-X21-C-X11 |  |
| VdSCP106 | X52-C-X79-C-X4-C-X3-C-X20-C-X3-C-X89-C-X3 |  |
| VdSCP108 | X22-C-X5-C-X36-C-X5-C-X31-C-X5-C-X36-C-X5-C-X35-C-X5-C-X12 |  |
| VdSCP109 | X71-C-X3-C-X8-C-X6-C-X1-C-X7-C-X6-C-X3-C-X1-C-X1-C-X17 |  |
| VdSCP110 | X50-C-X7-C-X7-C-X16-C-X50-C-X13 |  |
| VdSCP111 | X28-C-X3-C-X8-C-X3-C-X19-C-X4-C-X73-C-X9-C-X29-C-X3-C-X12-C-X2-C-X2 |  |
| VdSCP115 | X8-C-X4-C-X11-C-X19-C-X19-C-X2-C-X14-C-X1-C-X28-C-X25 |  |
| VdSCP117 | X17-C-X39-C-X19-C-X20-C-X20-C-X18-C-X22 |  |
| VdSCP118 | X31-C-X20-C-X34-C-X45-C-X20 |  |
| VdSCP119 | X8-C-X3-C-X9-C-X197-C-X1-C-X13-C-X2-C-X17-C-X58 |  |
| VdSCP121 | X226-C-X8-C-X32-C-X22-C-X67-C-X2-C-X2 |  |
| VdSCP122 | X10-C-X49-C-X18-C-X35-C-X21-C-X22-C-X16 |  |
| VdSCP123 | X19-C-X20-C-X91-C-X14-C-X7-C-X22-C-X30 |  |
| VdSCP124 | X6-C-X3-C-X26-C-X5-C-X41-C-X8-C-X18-C-C-X23-C-X5-C-X51 |  |
| VdSCP126 | X118-C-X31-C-X28-C-X9-C-X1 |  |
| VdSCP127 | X18-C-X9-C-X32-C-X59-C-X20-C-X3-C-X9 |  |

Note: C, cysteine; X, random amino acid residue; SCRs, small cysteine rich proteins. VdSCPs in gray color were excluded from small cysteine rich proteins.
